# Transcutaneous auricular vagus nerve stimulation improves depressive-like behaviors in CUMS rats through regulation of gut microbiome, serum metabolites, and immune factors

**DOI:** 10.3389/fmicb.2026.1820578

**Published:** 2026-07-01

**Authors:** Chaoren Tan, Meng Qiao, Yue Ma, Xiaoling Wang, Min Xing, Shiyue Sun, Yinan Shi, Yingying Wang, Jiliang Fang, Yongsheng Yang

**Affiliations:** 1 Institute of Acupuncture and Moxibustion, China Academy of Chinese Medical Sciences, Beijing, China; 2 Institute of Basic Research in Clinical Medicine, China Academy of Chinese Medical Sciences, Beijing, China; 3 Guang’anmen Hospital, China Academy of Chinese Medical Sciences, Beijing, China; 4 Department of Traditional Chinese Medicine, National Center for Children's Health, Beijing Children's Hospital, Capital Medical University, Beijing, China

**Keywords:** *Bifidobacterium animalis*, depression, gut microbiota, indole-3-lactic acid, *Lactobacillus murinus*, metabolomics, transcutaneous auricular vagus nerve stimulation

## Abstract

**Background:**

Depression is associated with microbiota-gut-brain (MGB) axis dysregulation. Transcutaneous auricular vagus nerve stimulation (taVNS) has shown antidepressant effects and modulated gut microbiota, but its potential to alleviate depression specifically via modulation of the MGB axis remains largely unexplored.

**Methods:**

Rats subjected to chronic unpredictable mild stress (CUMS) received taVNS for 3 weeks. We assessed depressive-like behaviors, gut microbiota, plasma metabolism, and inflammatory marker levels. Pearson correlation analyses examined relationships among these factors.

**Results:**

taVNS significantly improved depressive behaviors in CUMS rats. It shifted gut microbiota composition, enriching beneficial *Lactobacillus murinus*, *Bifidobacterium animalis*, and Prevotellaceae while reducing harmful *Bacteroidales* and Romboutsia. Metabolomics revealed taVNS modulated plasma metabolism, especially metabolism of cofactor/vitamin, sphingolipid metabolism, amino and organic acid metabolism, increasing the levels of indole-3-lactic acid (ILA), riboflavin, sphingosine-1-phosphate (S1P), sphinganine-1-phosphate (Sa1P) and sphingosine (SP), and creatine. taVNS also reduced blood, hippocampus and prefrontal cortex inflammation. Pearson correlation analysis showed that alleviation of depressive behaviors positively correlated with *Lactobacillus murinus*, *Bifidobacterium animalis*, and plasma ILA, riboflavin, S1P, Sa1P, SP, and creatine and all these parameters inversely associated with pro-inflammatory factors.

**Conclusion:**

These findings indicate that taVNS may alleviate depression by enriching *Lactobacillus murinus* and *Bifidobacterium animalis* to enhance biosynthesis of microbiota-derived metabolites (ILA, riboflavin) and modulate host plasma metabolites (S1P, Sa1P, SP, creatine), thereby attenuating systemic and neuroinflammatory processes.

## 1 Introduction

Major depressive disorder (MDD) is a prevalent mental health condition imposing a substantial global burden of severe disability, morbidity, and mortality. Current estimates indicate that approximately 280 million people worldwide are affected by depression (Vos et al., 2020), corresponding to a lifetime prevalence of approximately 20% (Malhi and Mann, 2018). Projections further suggest MDD will become the leading cause of the global disease burden by 2030 (GBD 2017 Disease and Injury Incidence and Prevalence Collaborators, 2018). While the monoamine hypothesis dominates current pathophysiology models (Zhang W. et al., 2021), antidepressants based on it achieve remission in only 50–70% of patients and are often limited by side effects, withdrawal, and recurrence (Locher et al., 2017; Cipriani et al., 2018). Consequently, other possible treatments for MDD are anticipated.

The microbiota-gut-brain (MGB) axis represents a critical bidirectional communication system involving the vagus nerve, immune mediators, neuroendocrine pathways, and gut microbiota-derived metabolites (Cryan et al., 2019). Substantial evidence implicates dysregulation of the MGB axis in the pathophysiology of MDD (Evrensel and Ceylan, 2015). Both clinical and preclinical studies consistently associate depression with gut microbiota dysbiosis. This dysbiosis drives significant alterations in the gut metabolome and contributes to gastrointestinal metabolic dysfunction (Yang et al., 2020). Crucially, fecal microbiota transplantation from MDD patients or depression-model rodents into healthy recipients is sufficient to induce depressive-like behaviors (Zheng et al., 2016; Liu P. et al., 2024). Conversely, probiotic interventions demonstrate efficacy in ameliorating these behaviors (Liu P. et al., 2024). Furthermore, an altered gut metabolome itself promotes depressive-like phenotypes in rodents (Li J. et al., 2019), while supplementation with specific microbiota-derived metabolites, alleviates depressive symptoms in animal models (Zhao M. et al., 2024). Collectively, these findings highlight the gut microbiota and its metabolic output as promising novel targets for the diagnosis and treatment of depression.

Transcutaneous auricular vagus nerve stimulation (taVNS) is a non-invasive neuromodulation technique that targets the auricular branch of the vagus nerve. Substantial clinical evidence supports its efficacy and safety as a treatment for MDD (Rong et al., 2016; Fang et al., 2016; Tan et al., 2023). Beyond MDD, taVNS demonstrates therapeutic potential for diverse depressive conditions, including treatment-resistant depression (Li X. J. et al., 2019; Kaczmarczyk et al., 2021), post-stroke depression (Liu C. et al., 2024), and peripartum-onset MDD (Deligiannidis et al., 2022). Furthermore, taVNS shows efficacy for depression comorbid with epilepsy (Zyr et al., 2025), chronic pain (Li et al., 2022), and acquired immune deficiency syndrome (Zou et al., 2025), as well as for primary gut-related disorders (Liu T. et al., 2025). Given the vagus nerve’s established role as a critical bidirectional conduit for gut-brain communication (Tan et al., 2022), research into taVNS mechanisms extends beyond its modulation of neuronal activity and functional connectivity within the brain to include its impact on the gut-brain axis (Faraji et al., 2025). Recent clinical studies indicate that taVNS modulates gut microbiota composition, metabolic profiles, and alleviates symptoms like constipation and abdominal pain in patients with constipation-predominant irritable bowel syndrome (Shi et al., 2021; Liu et al., 2024b). Preclinically, taVNS enhances gastrointestinal motility (increasing fecal pellet number, water content, and transit) in irritable bowel syndrome with constipation model mice and restores the abundance of beneficial genera, specifically *Lactobacillus* and *Bifidobacterium* (Liu et al., 2024a). Similarly, vagus nerve stimulation alleviates motor deficits, gastrointestinal dysfunction, intestinal and neuroinflammation, and mitigates gut microbiota dysbiosis in rodent models (Wang Y. et al., 2024). Importantly, activation of parasympathetic vagal neurons reversed the effects of stress on the gut microbiome and immunity through Brunner’s glands (Chang H. et al., 2024). While prior work indicates that taVNS modulates the gut microbiome, its specific effects on gut microbiota composition and metabolism within the context of depression remain poorly characterized.

Consequently, the present study employed the chronic unpredictable mild stress (CUMS) rat model of depression (Li et al., 2021) to investigate the impact of taVNS on depressive-like behaviors. Using an integrated microbiota and metabolomics approach, we elucidated the underlying mechanisms of taVNS action. Furthermore, we also explored the relationship between changed gut microbiota, plasma metabolites, pro-inflammatory factors and depressive-like behaviors. Our findings provide novel insights into how taVNS ameliorates depression through modulation of the MGB axis.

## 2 Materials and methods

### 2.1 Animals

Twenty five male Sprague–Dawley rats (8 weeks old, 180–220 g) were purchased from Sibeifu (Beijing) Biotechnology Co., Ltd. (production license number: SCXK (Jing) 2019-0010). They were housed under standard conditions (20–26 °C, 12-h light/dark cycle, lights on 08:00–20:00) with ad libitum access to food and water. All procedures were approved by the Medical Ethics Committee of the Institute of Acupuncture and Moxibustion, Chinese Academy of Traditional Chinese Medicine (Approval number: D2022-06-12-4) and followed the NIH Guide for the Care and Use of Laboratory Animals.

### 2.2 Experiment design

**Figure 1:**
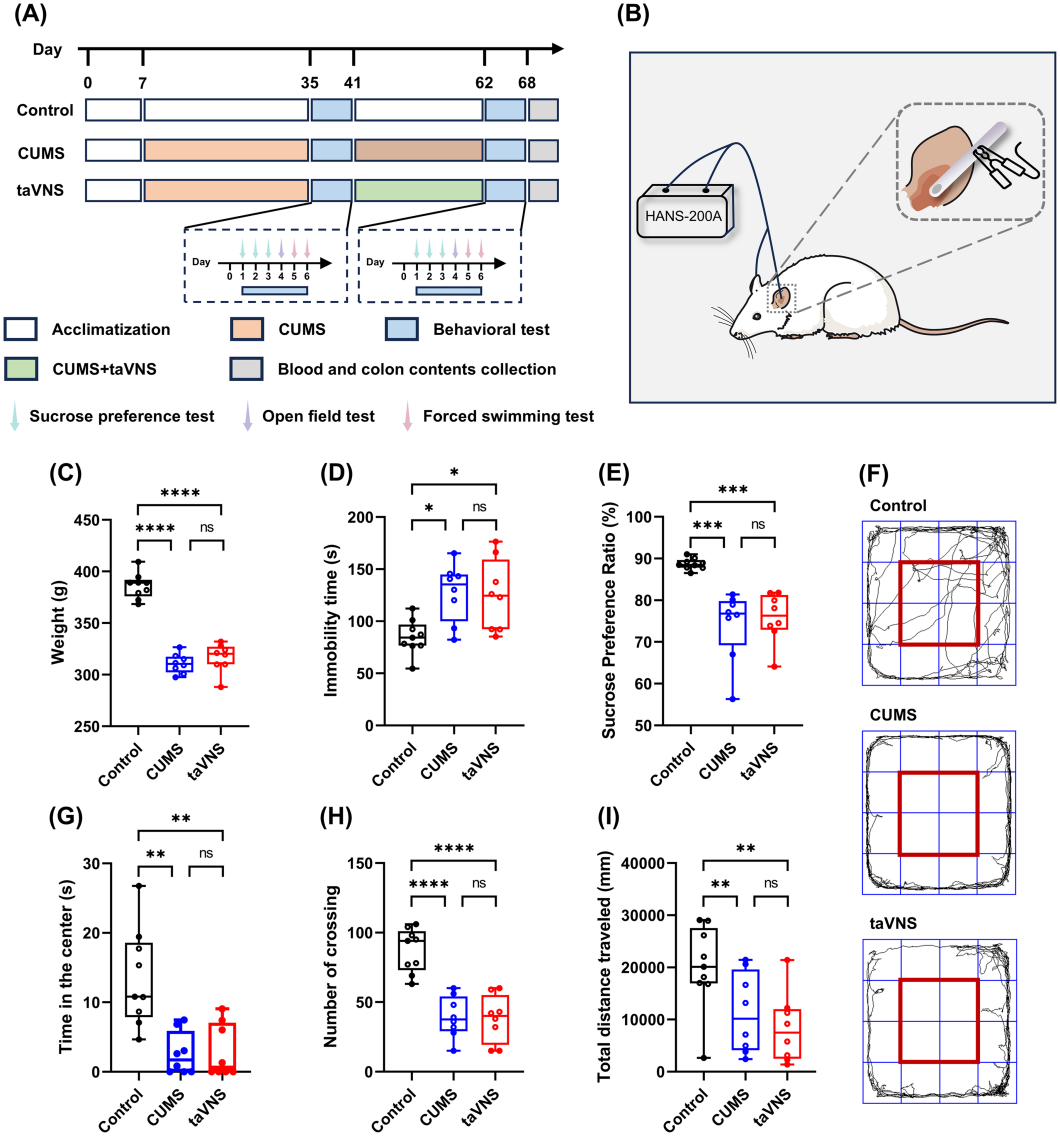
The schematic representation of taVNS treatment in depression rats and their behavioral tests after CUMS modeling. **(A)** The diagram of taVNS treatment procedures. **(B)** The diagram of transcutaneous auricular vagus nerve stimulation. The taVNS targets the bilateral auricular concha, which is innervated by the auricular branch of the vagus nerve. **(C)** The effects of CUMS on the body weight. **(D)** The effects of CUMS on immobility time in the FST. **(E)** The effect of CUMS on the percentage of sucrose consumption in the SPT. **(F)** The representative movement traces of rats in different groups in the OFT. **(G)** Comparison of the time spent in center in the OFT. **(H)** Comparison of number of squares crossing in the OFT. **(I)** Comparison of the distance traveled in the OFT. Data were expressed as the mean ± SD (*n* = 9 for Control group; *n* = 8 for CUMS and taVNS groups). *p < 0.05, **p < 0.01, ***p < 0.001, ****p < 0.0001. ns, no significant.

After acclimatization, rats were randomized into three groups (Control: *n* = 9; CUMS: *n* = 8; taVNS: *n* = 8). Rats in the Control group were kept in their own cages (3 or 4 rats per cage) except for behavior tests. The CUMS and taVNS groups underwent 28 days of CUMS in single cages. Following 21 days of taVNS treatment concurrent with stress (CUMS group stress only), behavioral tests including sucrose preference (SPT), open-field (OFT), and forced swim (FST) were performed sequentially at 24 h intervals on Days 36 and 63. Rats were sacrificed 24 h post-final test for blood and colon collection. Timeline details are in Figure 1A.

### 2.3 Chronic unpredictable mild stress paradigm

CUMS was performed as described with modifications (Li et al., 2021). Rats were singly housed and exposed daily for 28 days to one of seven unpredictable stressors (physical restraint: 2 h; altered light cycle: 24 h; food deprivation: 24 h; water deprivation: 24 h; tail clamping: 60 s; cold swim (4 °C): 5 min; soiled bedding: 24 h) in a randomized non-repeating sequence. Each stressor was applied four times.

### 2.4 Transcutaneous auricular vagus nerve stimulation and sham-taVNS protocol

After behavioral tests on day 41, taVNS rats received daily 30-min stimulation for 21 days. Under 1.5% isoflurane anesthesia (induced at 1–2%), auricular clamps positioned bilaterally on the concha (Figure 1B) were connected to a HANS-200A device (Nanjing Jisheng Medical Technology). The rats in the Control and CUMS groups received the sham-taVNS with the same anesthesia procedure as the taVNS group and auricular clamps positioned bilaterally on the concha but not connected to a HANS-200A device for 30-min sham- stimulation for 21 days. Stimulation parameters: dual-frequency (2/15 Hz alternating at 1-s intervals), 2 mA intensity, delivered daily between 10:00–12:00.

### 2.5 Behavior evaluation

#### 2.5.1 Sucrose preference test

The SPT assessed anhedonia. After 24 h adaptation to 1% sucrose and water (bottles alternated every 6 h), rats were water-deprived for 24 h. They were then given simultaneous access to both liquids for 2 h (bottle positions counterbalanced midway). Sucrose preference (%) = sucrose intake/(sucrose + water intake).

#### 2.5.2 Open field test

The OFT evaluated locomotion and anxiety. Rats were placed peripherally in a black, square arena (100 × 100 × 40 cm) with a defined center zone (50 cm diameter). Movements were tracked automatically for 5 min. Parameters assessed including center time, total distance traveled, and line crossings.

#### 2.5.3 Forced swimming test

The FST quantifies behavioral despair. Twenty-four hours prior to formal testing, rats underwent a 10-min habituation swim (22–24 °C). The next day, they were tested for 5 min under identical conditions. Immobility (floating with minimal movement) during the test was quantified by automated tracking.

### 2.6 16S rRNA sequencing of the gut microbiome

Genomic DNA was extracted from colonic contents using the CTAB or SDS method, and its purity and concentration were assessed by agarose gel electrophoresis. DNA was diluted to 1 ng/μL with sterile water, and the V4 region of the bacterial 16S rRNA gene was amplified using barcoded primers 515F and 806R with Phusion^®^ High-Fidelity PCR Master Mix with GC Buffer (New England Biolabs, United States). PCR products were verified on 2% agarose gels, purified using magnetic beads, pooled in equimolar amounts, and recovered by gel extraction (QIAquick Gel Extraction Kit, Qiagen, Germany). Sequencing libraries were constructed using the TruSeq^®^ DNA PCR-Free Sample Preparation Kit (Illumina, United States), quantified by Qubit and qPCR, and sequenced on the NovaSeq 6000 platform (Illumina, United States) with paired-end reads. Raw reads were demultiplexed and adapter-trimmed, then quality-filtered using fastp (v0.22.0) with the following criteria: removal of reads containing ≥15 N bases, reads with >50% low-quality bases (*Q* ≤ 20), reads with an average quality score <20 in a 4-base sliding window, poly-G tails, and reads shorter than 150 bp. High-quality paired-end reads were merged using FLASH (v1.2.11) to generate clean tags, and chimeric sequences were detected and removed using vsearch (v2.22.1) to obtain effective tags. ASVs were generated using DADA2 (v1.26.0) or Deblur (v1.1.1) in QIIME 2 (v2023.2). Taxonomic annotation was performed using Mothur (v1.48) against the SILVA 138.1 SSU rRNA database with a confidence threshold of 0.8–1.0 at the phylum to species levels. Multiple sequence alignment was conducted using MAFFT (v7.520). Data were rarefied to the minimum sequencing depth for normalization.

Alpha diversity indices (observed ASVs, Chao1, ACE, Shannon, and Simpson) were calculated using the phyloseq (v1.40.0) and vegan (v2.6.2) packages in R (v4.2.0). Beta diversity was evaluated using weighted UniFrac distances computed with phyloseq (v1.40.0), and visualized by principal component analysis and non-metric multidimensional scaling (NMDS) using phyloseq (v1.40.0). Linear discriminant analysis effect size (LEfSe) was performed using LEfSe (v1.1.2) with default settings (LDA score > 3.5 and *p* < 0.05). Metastats analysis was conducted using Mothur at the order, genus, and species levels with permutation testing; *p* values were corrected using the Benjamini-Hochberg false discovery rate method to obtain *q* values.

### 2.7 Metabolomics analysis

Frozen samples were thawed on ice, vortexed, and extracted with acetonitrile:methanol (1:4, v/v) containing internal standards. After vortexing and centrifugation (12,000 rpm, 10 min, 4 °C), the supernatant was collected, re-centrifuged after chilling at −20 °C, and subjected to LC–MS analysis. Chromatographic separation was performed on a Waters ACQUITY Premier HSS T3 column (1.8 μm, 2.1 × 100 mm) at 40 °C with a gradient elution of 0.1% formic acid in water (A) and acetonitrile (B): 5–20% B (2 min), 60% B (3 min), 99% B (1 min, held 1.5 min), then returned to 5% B (0.1 min, held 2.4 min). Flow rate: 0.4 mL/min; injection volume: 4 μL. Both positive and negative ion modes were used. MS parameters: GAS1/GAS2, 50 psi; CUR, 25 psi; TEM, 550 °C; DP, ±60 V; ISVF, +5,000/−4,000 V; mass range, 50–1,000 Da; accumulation time, 200 ms. Raw data were converted to mzXML format using ProteoWizard, and peaks were extracted, aligned, and corrected using XCMS. Metabolites with detection rate <50% in any group were excluded. Metabolite identification was performed by searching against in-house, public, AI, and metDNA databases. PCA was conducted in R after unit variance scaling. Differential metabolites were identified by VIP > 1 (from OPLS-DA) and *p* < 0.05 (Student’s *t*-test for two groups; ANOVA for multiple groups) using MetaboAnalystR, with 200 permutations to avoid overfitting. KEGG pathway enrichment was analyzed by hypergeometric test (*p* < 0.05).

Short-chain fatty acids (SCFAs) in cecal content were quantified using GC–MS/MS (Agilent 8890B-7000D). Briefly, 20 mg of homogenized sample was extracted with 1,000 μL of 0.5% (v/v) phosphoric acid via vortexing (10 min) and sonication (5 min), followed by centrifugation (12,000 rpm, 10 min, 4 °C). The supernatant (100 μL) was mixed with 500 μL methyl tert-butyl ether (MTBE) containing internal standards, re-extracted, and centrifuged. The organic layer (200 μL) was subjected to GC–MS/MS analysis. Chromatographic separation was achieved on a DB-FFAP capillary column (30 m × 0.25 mm, 0.25 μm) with helium carrier gas (1.2 mL/min). The temperature program was: 50 °C (1 min) → 220 °C at 18 °C/min → hold 5 min. Key MS parameters included: MRM detection mode, injector temperature 250 °C, transfer line 230 °C, and quadrupoles at 150 °C. Quantification utilized a 13-point external calibration curve (0.005–20 μg/mL) with internal standard correction. Method validation confirmed linearity (*R*^2^ > 0.99 for all analytes), precision (CV ≤ 15%), and accuracy (85–115% recovery). Results were expressed as mg/g wet weight.

### 2.8 Enzyme linked immunosorbent assay

Concentrations of tumor necrosis factor alpha (TNF-α), interleukin 1 beta (IL-1β), and interleukin 6 (IL-6) in hippocampal and prefrontal cortex (PFC) tissue homogenates and serum, as well as serum corticosterone levels, were quantified using specific commercial enzyme-linked immunosorbent assay (ELISA) kits according to the manufacturers’ protocols. All samples were analyzed in duplicate. Specific kits were employed as follows: Rat TNF-α: Kit No. CSB-E11987r (Cusabio Technology LLC, Wuhan, China); Rat IL-1β: Kit No. CSB-E08055r (Cusabio Technology LLC, Wuhan, China); Rat IL-6: Kit No. CSB-E04640r (Cusabio Technology LLC, Wuhan, China); Rat Corticosterone: Kit No. ADI-900-097 (Enzo Life Sciences, Farmingdale, NY, United States). Absorbance was measured at 450 nm using a microplate reader (BioTek Synergy H1, United States). Analyte concentrations were interpolated from standard curves and normalized to total protein content (determined by BCA assay) for tissue samples.

### 2.9 Immunofluorescence staining and microglial quantification

Coronal brain sections (20 μm) encompassing the dorsal hippocampus were fixed in 4% paraformaldehyde (15 min), permeabilized with 0.3% Triton X-100 (10 min), and blocked with 5% normal donkey serum (1 h). Sections were incubated with primary antibodies at 4 °C overnight: Iba1 (microglia marker): Rabbit polyclonal anti-Iba1 (1:500; Fujifilm Wako #019-19741, Japan); CD68 (phagocytic microglia marker): Mouse monoclonal anti-CD68 (1:200; Bio-Rad #MCA341GA, UK). After PBS washes for 3 times (5 min per time), species-matched secondary antibodies were applied for 2 h at room temperature in the dark: Donkey anti-rabbit IgG Alexa Fluor 488 (1:500; Jackson ImmunoResearch #711-545-152); Donkey anti-mouse IgG Alexa Fluor 594 (1:500; Jackson ImmunoResearch #715-585-150); Sections were counterstained with 4′,6-diamidino-2-phenylindole (DAPI, 1:1000, Sigma-Aldrich, D9542) for 10 min to visualize nuclei, washed, and mounted with antifade mounting medium (ProLong Gold, Invitrogen, P36930).

Quantitative analysis was performed on hippocampal CA1 and dentate gyrus (DG) subregions using a confocal microscope (Zeiss LSM 900, 40 × objective). For each region, five non-overlapping fields per animal were captured. Iba1^+^ cells were identified by DAPI^+^ nuclei with cytoplasmic Iba1 signal. The density of Iba1^+^ cells (cells/mm^2^) and the proportion of activated microglia (Iba1^+^CD68^+^/Iba1^+^) were calculated using ImageJ (v1.53) with threshold-based segmentation and colocalization analysis.

### 2.10 Statistical analysis

Data were presented as mean ± SD. Continuous data were assessed for normality using the Shapiro–Wilk test; data failing normality or homogeneity of variance assumptions were analyzed using the Non-parametric test. Group differences in body weight, behavioral test results, Tax4Fun2 functional prediction, plasma metabolites, cell counts, and inflammatory cytokine levels were analyzed by one-way ANOVA with Tukey’s *post-hoc* test. Beta diversity index differences among groups were assessed by Kruskal-Wallis test. For Metastats analysis, differentially abundant taxa between groups were identified by *T*-test. The correlation heat map was generated using ChiPlot.^1^ All analyses and other graphs were generated using SPSS 20.0, R (v4.2.0), and GraphPad Prism 8, with *p* < 0.05 considered significant.

## 3 Results

### 3.1 CUMS modeling decreased body weight and induced depressive-like behaviors in rats

Following CUMS modeling, both CUMS and taVNS groups exhibited significantly reduced body weight (Figure 1C), increased immobility time in the FST (Figure 1D), and decreased sucrose preference (Figure 1E) compared to Controls. OFT measures (center time, crossings, distance traveled) were also significantly lower in both groups (Figures 1F–I).

### 3.2 taVNS intervention ameliorated depressive-like behaviors in CUMS rats

**Figure 2:**
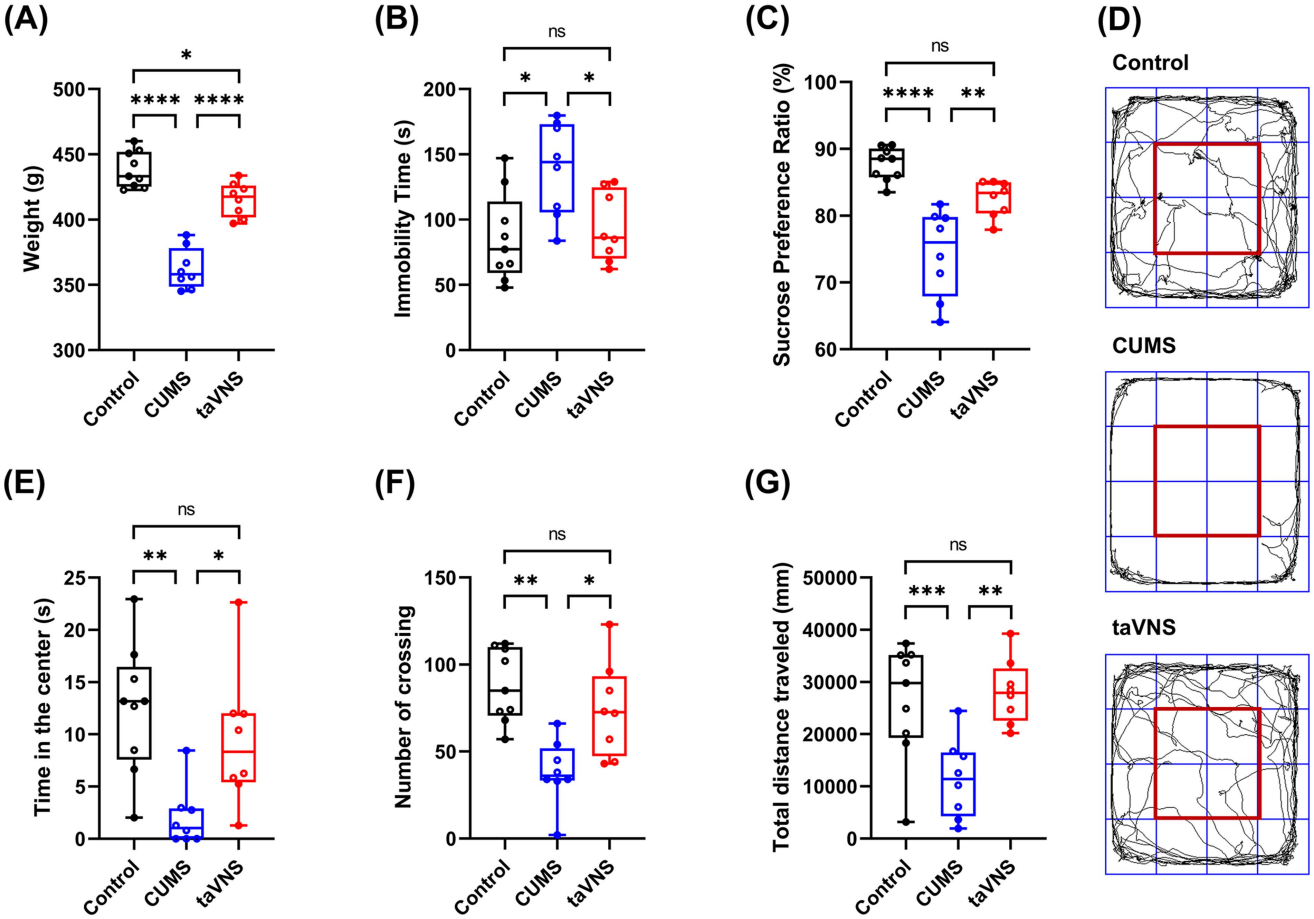
taVNS ameliorated depressive-like behaviors in CUMS treated rats. **(A)** The effects of taVNS on the body weight. **(B)** The effects of taVNS on immobility time in the FST. **(C)** The effect of taVNS on the percentage of sucrose consumption in the SPT. **(D)** The representative movement traces of rats in different groups in the OFT. **(E)** Comparison of the time spent in center in the OFT. **(F)** Comparison of number of squares crossing in the OFT. **(G)** Comparison of the distance traveled in the OFT. Data were expressed as the mean ± SD (*n* = 9 for Control group; *n* = 8 for CUMS and taVNS groups). *p < 0.05, **p < 0.01, ***p < 0.001, ****p < 0.0001. ns, no significant.

Post taVNS intervention (Figures 2A–G), the CUMS group maintained significant reductions in body weight, sucrose preference, and OFT parameters, alongside prolonged immobility time in the FST. However, taVNS intervention effectively normalized these CUMS-induced deficits: body weight recovered near control levels by day 69, and all other behavioral indicators returned to levels comparable to the Control group.

### 3.3 taVNS modulated the composition of gut microbiota and microbial functions in rats exposed to CUMS

**Figure 3:**
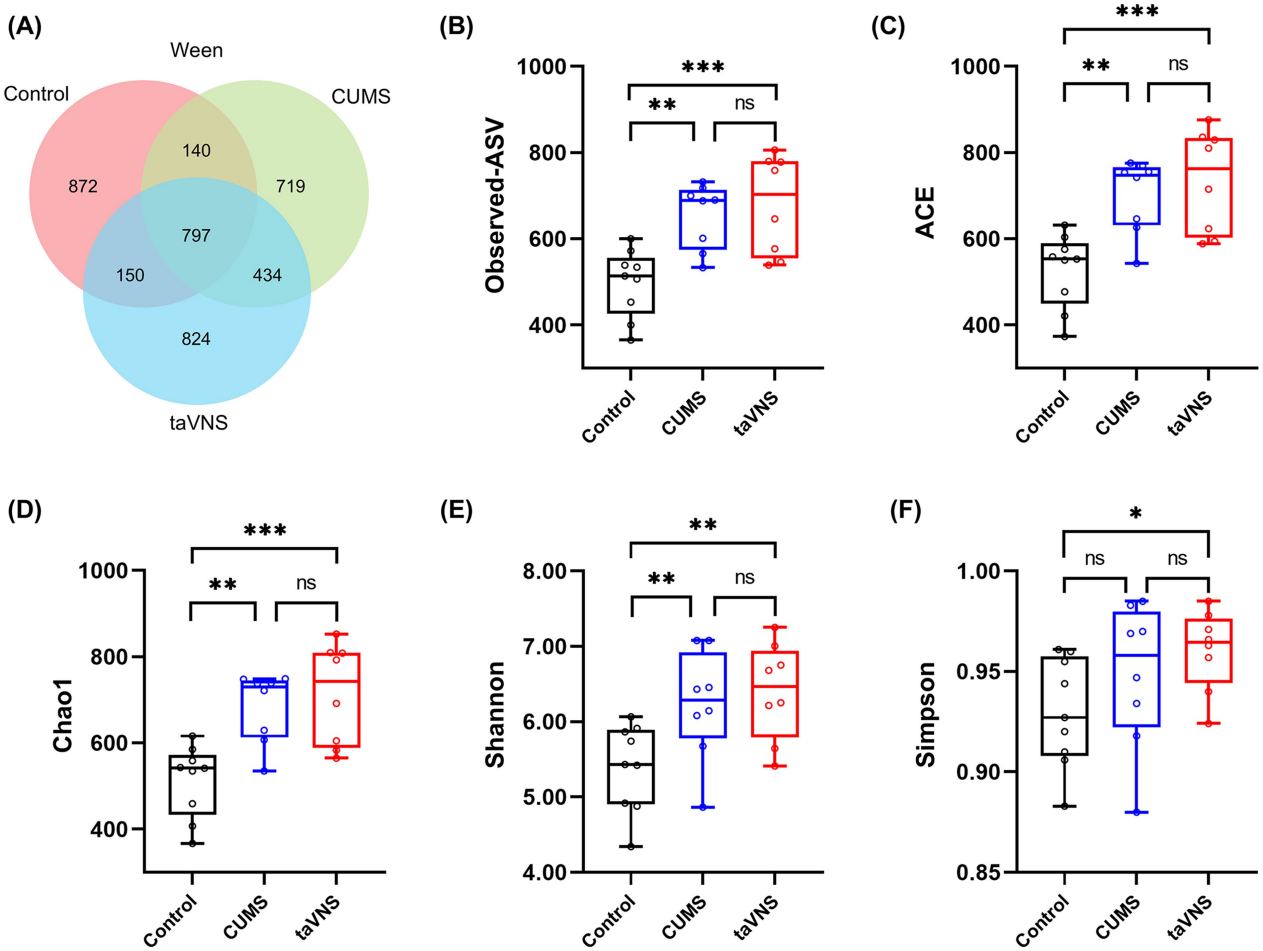
Alpha diversity of gut microbial in rats of each group (*n* = 9 for Control group; *n* = 8 for CUMS and taVNS groups). **(A)** The number of common and unique AVSs among the 3 groups is displayed by the Venn diagram. **(B–F)** Alpha diversity analysis indices, including the **(B)** observed-ASVs, **(C)** Ace index, **(D)** Chao1 index, **(E)** Shannon index, and **(F)** Simpson index. *p < 0.05, **p < 0.01, ***p < 0.001, ns, no significance.

**Figure 4:**
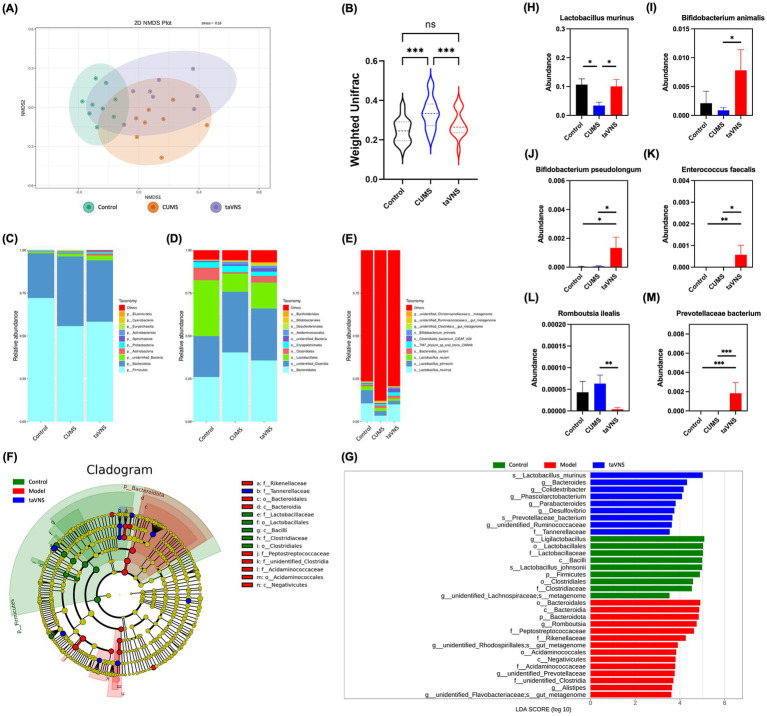
taVNS altered gut microbiome composition in rats exposed to CUMS (*n* = 9 for Control group; *n* = 8 for CUMS and taVNS groups). **(A)** Non-Metric Multi-Dimensional Scaling analysis based on ASVs of the 3 groups. **(B)** The beta-diversity index of gut microbiota among the 3 groups evaluated by Weighted Unifrac based on ASVs. **(C)** Stacked impact diagram of the top 10 microorganisms ranked by relative abundance at the phylum level. **(D)** Stacked impact diagram of the top 10 microorganisms ranked by relative abundance at the order level. **(E)** Stacked impact diagram of the top 10 microorganisms ranked by relative abundance at the species level. **(F)** Cladogram analysis among the 3 groups, with the center point representing the root of the tree (bacteria) and each circle representing the next lower taxonomic level. **(G)** LEfSe analysis histograms (LDA > 3.5, *p* < 0.05). Comparisons of the relative species abundance of *Lactobacillus murinus* **(H)**, *Bifidobacterium animalis* **(I)**, *Bifidobacterium pseudolongum* **(J)**, *Enterococcus faecalis* **(K)**, *Romboutsia ilealis* **(L)** and *Prevotellaceae bacterium* **(M)** among Control, CUMS and taVNS group. *p < 0.05, **p < 0.01, ***p < 0.001. ns, no significant.

Given the reported link between gut microbiome alterations and depressive-like behaviors, we assessed taVNS effects using 16S rDNA sequencing of colon contents. ASV counts differed significantly among groups (Control: 2059; CUMS: 2090; taVNS: 2205; Figures 3A,B). Alpha diversity indices (ACE, Chao1, Shannon, Simpson; Figures 3C–F) also differed significantly. CUMS and taVNS groups showed higher ACE, Chao1 (richness), and taVNS group showed higher Simpson (diversity) indices vs. Control, but no difference existed between CUMS and taVNS. Beta diversity analysis (weighted UniFrac, NMDS; Figures 4A,B) revealed distinct clustering: the Control and taVNS groups exhibited similar beta diversity, both significantly different from the CUMS group.

Taxonomically, *Firmicutes*, *Bacteroidota*, and *Actinobacteria* dominated at the phylum level. CUMS increased *Bacteroidota* abundance while decreasing *Firmicutes* abundance and the *Firmicutes*/*Bacteroidota* ratio; taVNS partially reversed these changes (Figure 4C; Supplementary Figure S1). Similar reversal patterns were observed at lower taxonomic levels: CUMS altered abundances of orders (e.g., increased *Bacteroidales* and decreased *Lactobacillales*, *Bifidobacteriales*, *Clostridiales*; Figure 4D) and species (e.g., decreased *Lactobacillus murinus*, *Bifidobacterium animalis*; Figure 4E), which were ameliorated by taVNS.

LEfSe analysis was conducted to identify the significantly influential taxa among groups. The results showed that CUMS enriched genera like *o*_*Bacteroidales* and g_Romboutsia, while taVNS enriched *g_Lactobacillus murinus* and *g_Prevotellaceae bacterium* (Figures 4F,G). Direct comparison (Metastats) confirmed taVNS significantly increased beneficial taxa (e.g., orders *Bifidobacteriales*, *Eubacteriales*; genera *Bifidobacterium*, *Ligilactobacillus*; species *Lactobacillus murinus*, *Bifidobacterium animalis*, *Bifidobacterium pseudolongum*, *Enterococcus faecalis*, *Prevotellaceae bacterium*) and decreased others (e.g., orders *Micrococcales*, *Rhizobiales*; genera *Rothia*, *Streptococcus*, *Monoglobus*; species *Romboutsia ilealis*) compared to CUMS (Figures 4H–M; Supplementary Tables S1, S2).

Collectively, these results demonstrate taVNS effectively restored CUMS-induced gut dysbiosis, normalizing microbial composition toward Control levels.

**Figure 5:**
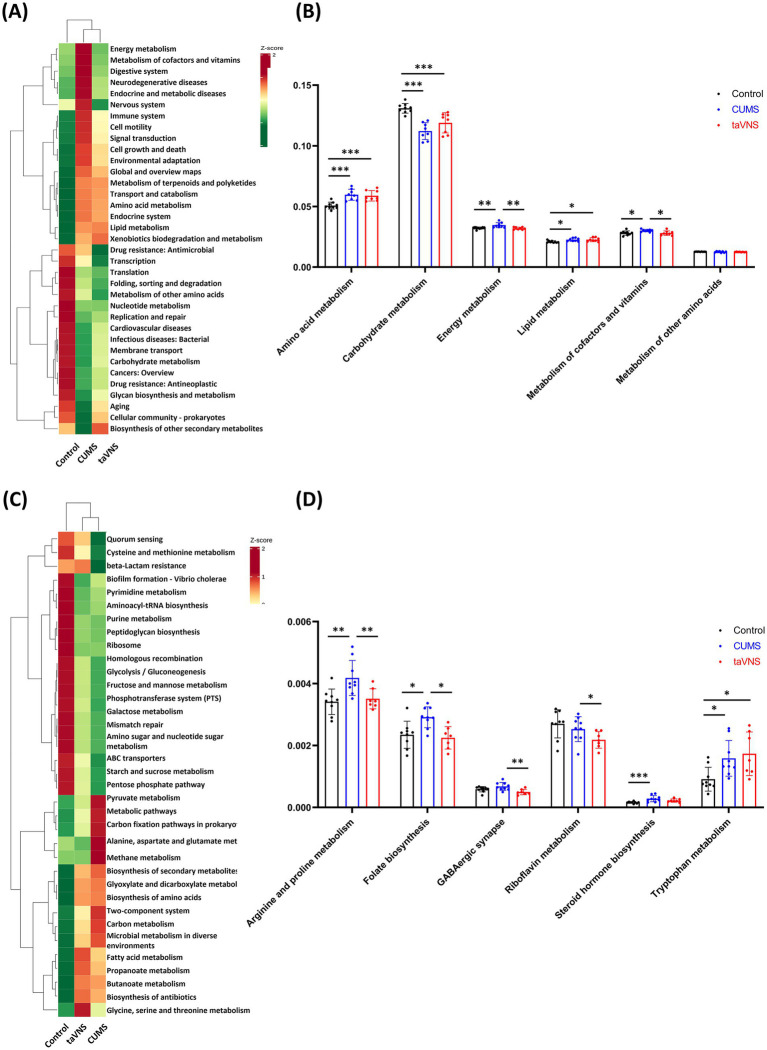
Alterations of gut microbial functions in taVNS group (*n* = 9 for Control group; *n* = 8 for CUMS and taVNS groups). **(A)** ASV-based Tax4Fun2 functional annotation clustered heatmap illustrates the variations of the microbial functions across the groups from the level 2 category. **(B)** The relative abundance of 6 pathways of the level 2 category. **(C)** ASV-based Tax4Fun2 functional annotation clustered heatmap illustrates the variations of the microbial functions across the groups from the level 3 category. **(D)** The relative abundance of 6 pathways of the level 3 category. *p < 0.05, **p < 0.01, ***p < 0.001.

To reveal potential host-microbe associations across groups, functional profiling of the gut microbiome was achieved using based on Tax4Fun2. Clustering heatmaps (Figures 5A,C) showed that taVNS partially reversed CUMS-induced alterations in microbial functions. At level 2, CUMS rats exhibited significant upregulation of Amino acid, Energy, and Lipid metabolism, and Metabolism of cofactors, alongside downregulated Carbohydrate metabolism versus controls (Figure 5B). taVNS significantly decreased Energy metabolism and Metabolism of cofactors and vitamins while increasing Carbohydrate metabolism versus CUMS (*p* = 0.05, Supplementary Table S3). At level 3, CUMS upregulated Arginine/proline metabolism, Folate biosynthesis, Steroid hormone biosynthesis, and Tryptophan metabolism (Figure 5D). taVNS significantly reduced Arginine/proline metabolism, Folate biosynthesis, and GABAergic synapse versus CUMS. Furthermore, taVNS partially reversed alterations in Steroid hormone biosynthesis (CUMS vs. taVNS, *p* = 0.65, Supplementary Table S4) and enhanced Riboflavin and Tryptophan metabolism changes induced by CUMS.

### 3.4 taVNS modulated the plasma metabolism of cofactor/vitamin, lipid metabolism and amino acid metabolism, while had limited impact on colonic short short-chain fatty acids concentrations in rats exposed to CUMS

Gut microbiota-derived SCFAs serve as critical mediators of bidirectional GBA communication (Hawkins et al., 2025). To investigate the effects of CUMS and taVNS on colonic SCFAs, a targeted metabolomic analysis was conducted to assess SCFA changes. Twelve SCFAs were quantified in the colonic contents of all rats. As shown in Supplementary Table S5, concentrations of nine SCFAs remained unaltered by CUMS or taVNS interventions. Administration of CUMS and taVNS elevated concentrations of decanoic acid, isobutyric acid, and isovaleric acid relative to the Control group.

**Figure 6:**
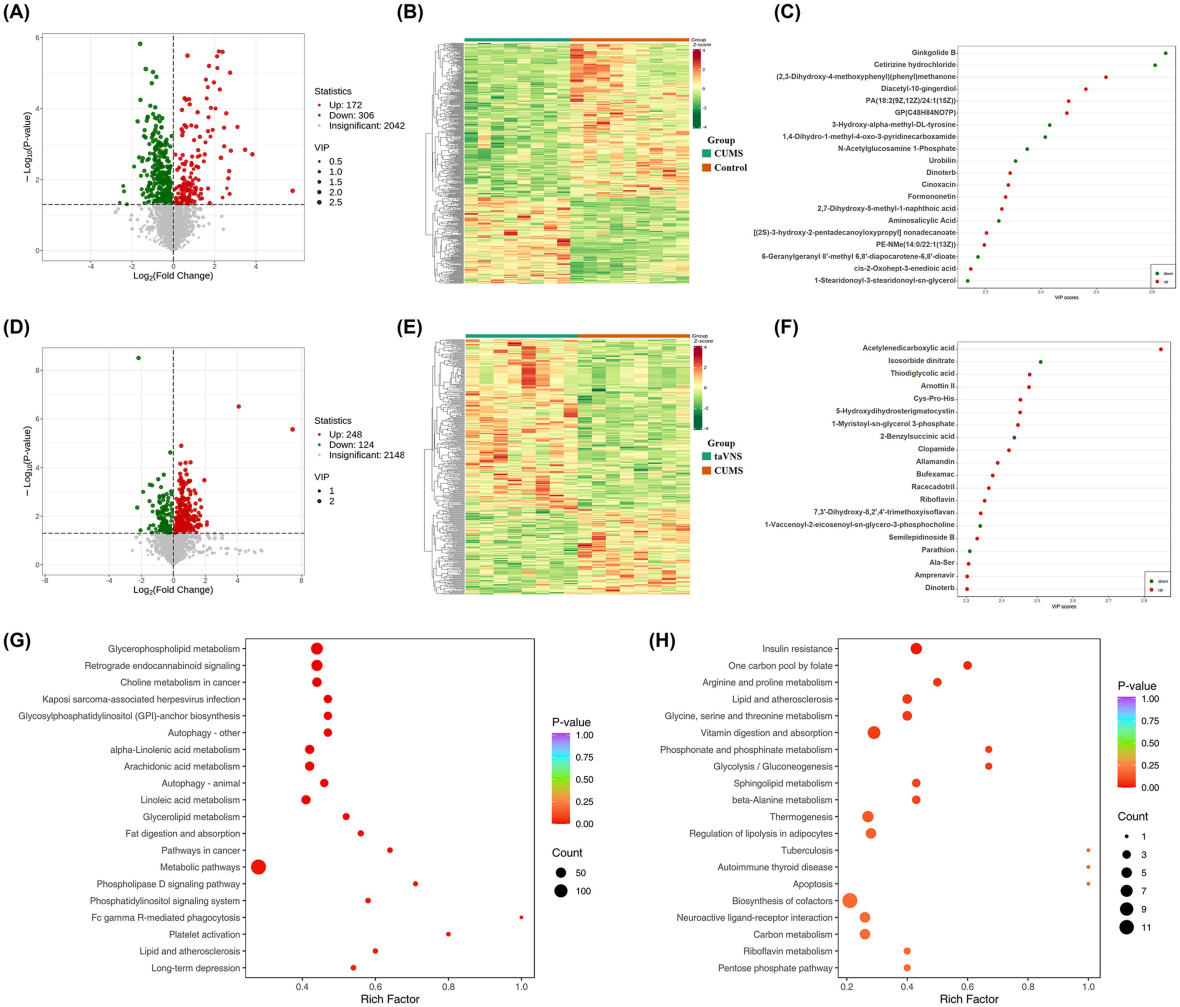
Plasma metabolomics comparisons between CUMS and both Control and taVNS groups (*n* = 9 for Control group; *n* = 8 for CUMS and taVNS groups). **(A)** The volcano plot identified different level of metabolites between the CUMS and Control group. **(B)** Clustered heat map of differential metabolites in KEGG pathways illustrating different plasma metabolism profile between the CUMS and Control group. **(C)** Plots of VIP values of differential metabolites (top 20 VIP values) between the CUMS and Control group. GP(C48H84NO7P), 1-(1Z-octadecenyl)-2-(4Z,7Z,10Z,13Z,16Z,19Z-docosahexaenoyl)-sn-glycero-3-phosphocholine. **(D)** The volcano plot identified different level of metabolites between the taVNS and CUMS group. **(E)** Clustered heat map of differential metabolites in KEGG pathways illustrating different plasma metabolism profile between the taVNS and CUMS group. **(F)** Plots of VIP values of differential metabolites (top 20 VIP values) between the taVNS and CUMS group. The x-axis represents the Rich Factor for each pathway, while the y-axis displays pathway names ordered by *p*-value (descending). The color gradient of the dots corresponds to the magnitude of the *p*-value, with redder hues indicating more significant enrichment. The size of the dots represents the number of enriched differential metabolites. **(G)** The KEGG differential metabolic pathways (top 20 *p*-values) between the CUMS and Control group. **(H)** The KEGG differential metabolic pathways (top 20 *p*-values) between the taVNS and CUMS group.

To investigate whether taVNS could modulate the plasma metabolism, untargeted metabolomics of plasma from rats of groups (*n* = 25 samples) were conducted. A total of 2,967 metabolites was detected. Compared to Control, CUMS showed 172 upregulated and 306 downregulated metabolites (Figures 6A,B). Among the top 20 VIP metabolites, 13 were upregulated (e.g., diacetyl-10-gingerdiol, dinoterb) and 9 downregulated (e.g., ginkgolide B, cetirizine HCl) in CUMS (Figure 6C). taVNS treatment, compared to CUMS, resulted in 248 upregulated and 124 downregulated metabolites (Figures 6D,E). Of the top 20 VIP metabolites, 16 were upregulated (e.g., riboflavin, acetylenedicarboxylic acid) and 4 downregulated (e.g., isosorbide dinitrate, 2-benzylsuccinic acid) in taVNS (Figure 6F).

KEGG pathway analysis of top 20 enriched pathways showed that CUMS vs. Control differences primarily involved Lipid metabolism (e.g., Glycerophospholipid, alpha-Linolenic acid, Arachidonic acid, Linoleic acid, Glycerolipid metabolism) and Nervous system/Signal transduction pathways (e.g., Retrograde endocannabinoid signaling, Phospholipase D, Phosphatidylinositol signaling). taVNS vs. CUMS differences were enriched in metabolism of Cofactor/vitamin (One carbon pool by folate, Riboflavin), Lipid metabolism (Sphingolipid metabolism, Lipid and atherosclerosis, Phosphonate and phosphinate metabolism) and Amino acid metabolism (Arginine and proline metabolism and Glycine, serine and threonine metabolism), (Figures 6G,H).

**Figure 7:**
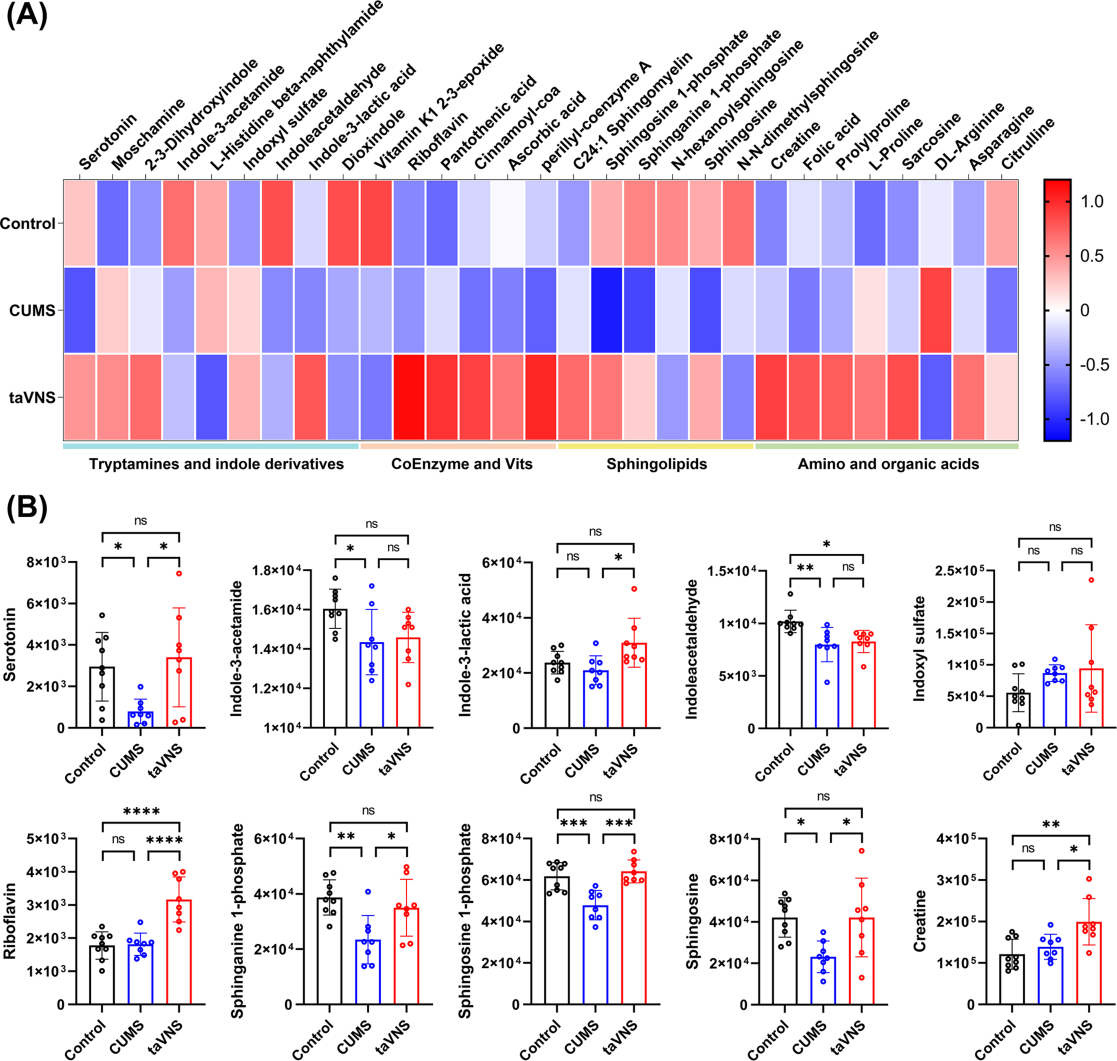
Heatmap of levels and one-way ANOVA analysis of tryptamines, indole derivations, co-enzyme and vitamins, sphingolipids, amino and organic acids across groups. **(A)** Heatmap of changes in tryptamines, indole derivations, co-enzyme and vitamins, sphingolipids, amino and organic acids. Vits, vitamins. **(B)** One-way ANOVA analysis of serotonin, indole-3-acetamide, indole-3-lactic acid, indoleacetaldehyde, indoxyl sulfate, riboflavin, sphingosine-1-phosphate, sphinganine-1-phosphate, sphingosine and creatine. *p < 0.05, **p < 0.01, ***p < 0.001, *p < 0.0001. ns, no significance.

Further one-way ANOVA analysis was conducted to investigate the effects of taVNS on tryptamines, indole derivations and metabolites related to metabolism of Cofactor/vitamin, Sphingolipid metabolism, Amino and organic acid metabolism (Figures 7A,B). The levels of serotonin, indole-3-acetamide, indoleacetaldehyde, sphingosine-1-phosphate (S1P), sphinganine-1-phosphate (Sa1P) and sphingosine (SP) were significantly reduced in CUMS rats. After taVNS treatment, the levels of serotonin, S1P, Sa1P and SP were significantly elevated. Additionally, taVNS significantly increased the levels of indole-3-lactic acid (ILA), riboflavin and creatine.

### 3.5 taVNS alleviated microglia activation in the hippocampal region and mitigated central and peripheral inflammatory cytokine responses in rats exposed to CUMS

**Figure 8:**
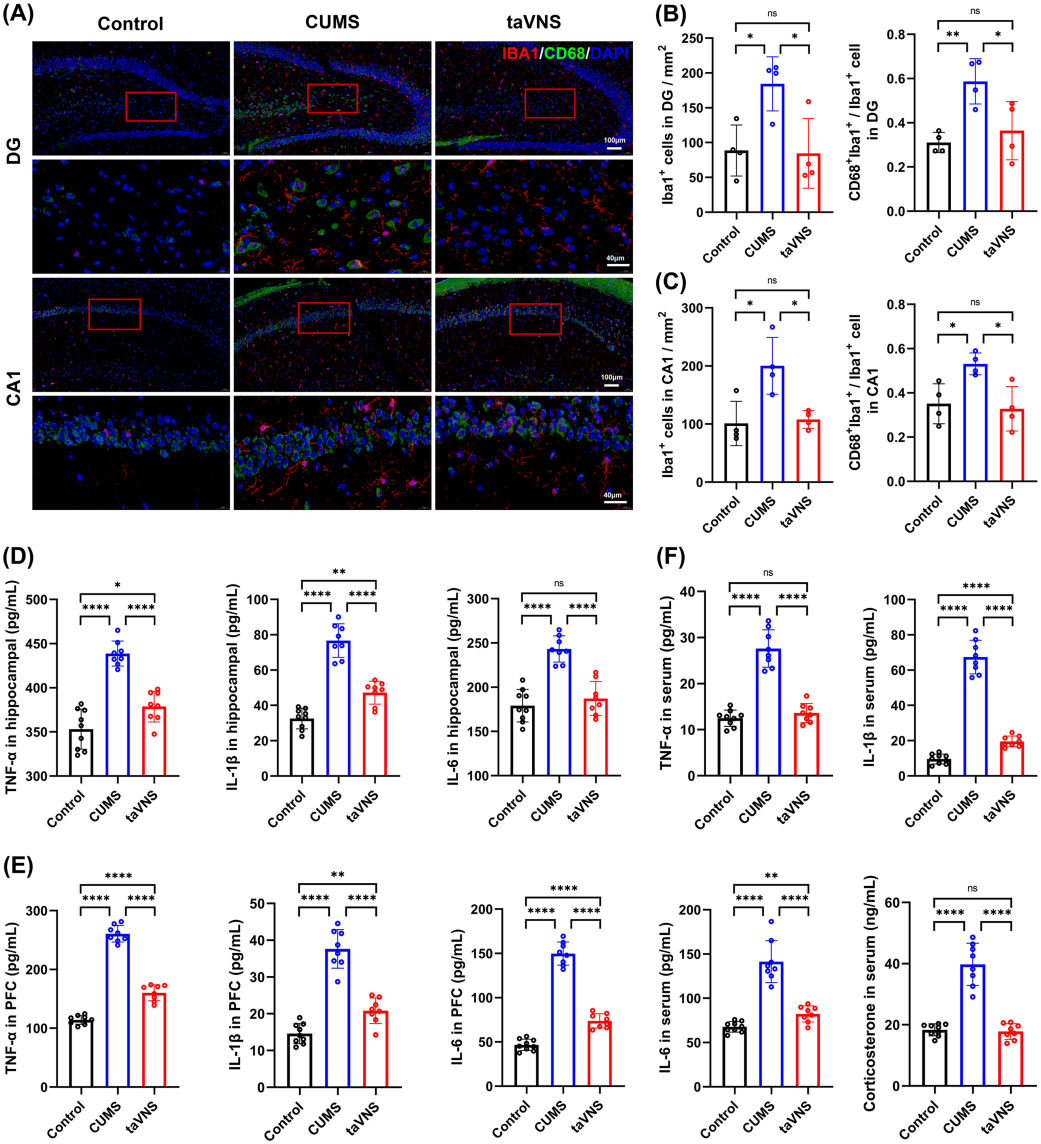
taVNS significantly ameliorated microglia activation in the hippocampal region and mitigated central and peripheral inflammatory cytokine responses in rats exposed to CUMS. **(A)** Representative immunofluorescence staining images of Iba1 + (red), CD68 + (green) and DAPI (blue) in DG and CA1. Scale bars for different magnifications are: 100 μm; 40 μm. Number of Iba1 positive cells and the ratio of CD68 and Iba1 double positive cells to Iba1 positive cells in DG **(B)** and CA1 **(C)** of rats across the 3 groups (*n* = 4). Concentration of TNF-α, IL-1β, and IL-6 in the hippocampal **(D)** and PFC **(E)** (*n* = 8). Concentration of TNF-α, IL-1β, IL-6 and corticosterone in the serum (*n* = 8) **(F)**. *p < 0.05, **p < 0.01, ****p < 0.0001. ns, no significant.

Inflammatory processes impact the behavioral and emotional aspects associated with depressive disorders (Yin et al., 2024). We investigated the effects of taVNS on microglial responses and serum pro-inflammatory cytokine responses induced by CUMS. Microglia and their activation state were identified by Iba1 and CD68 immunostaining, respectively. In the hippocampal CA1 and DG regions of CUMS rats, Iba1^+^ cells exhibited an activated morphology characterized by enlarged cell bodies and shortened processes. In contrast, Iba1^+^ cells in control rats displayed the typical resting morphology, with smaller cell bodies and thin, elongated processes (Figure 8A). CUMS significantly increased the number of Iba1^+^ cells in both CA1 and DG compared to the control group. This increase was significantly attenuated by taVNS treatment (Figures 8B,C). Furthermore, taVNS also significantly reduced the CUMS-induced elevation in the ratio of CD68^+^/Iba1^+^ cells to total Iba1^+^ cells in these regions (Figures 8B,C).

Compared to controls, CUMS rats exhibited significantly elevated levels of the pro-inflammatory cytokines TNF-α IL-1β, and IL-6 in the hippocampus, PFC, and serum. taVNS treatment significantly reduced these elevated cytokine levels (Figures 8D–F). Additionally, taVNS significantly ameliorated the CUMS-induced increase in serum corticosterone level (Figure 8F).

### 3.6 Relationships among depressive-like behaviors, microbial features, plasma metabolites and pro-inflammatory factors

**Figure 9:**
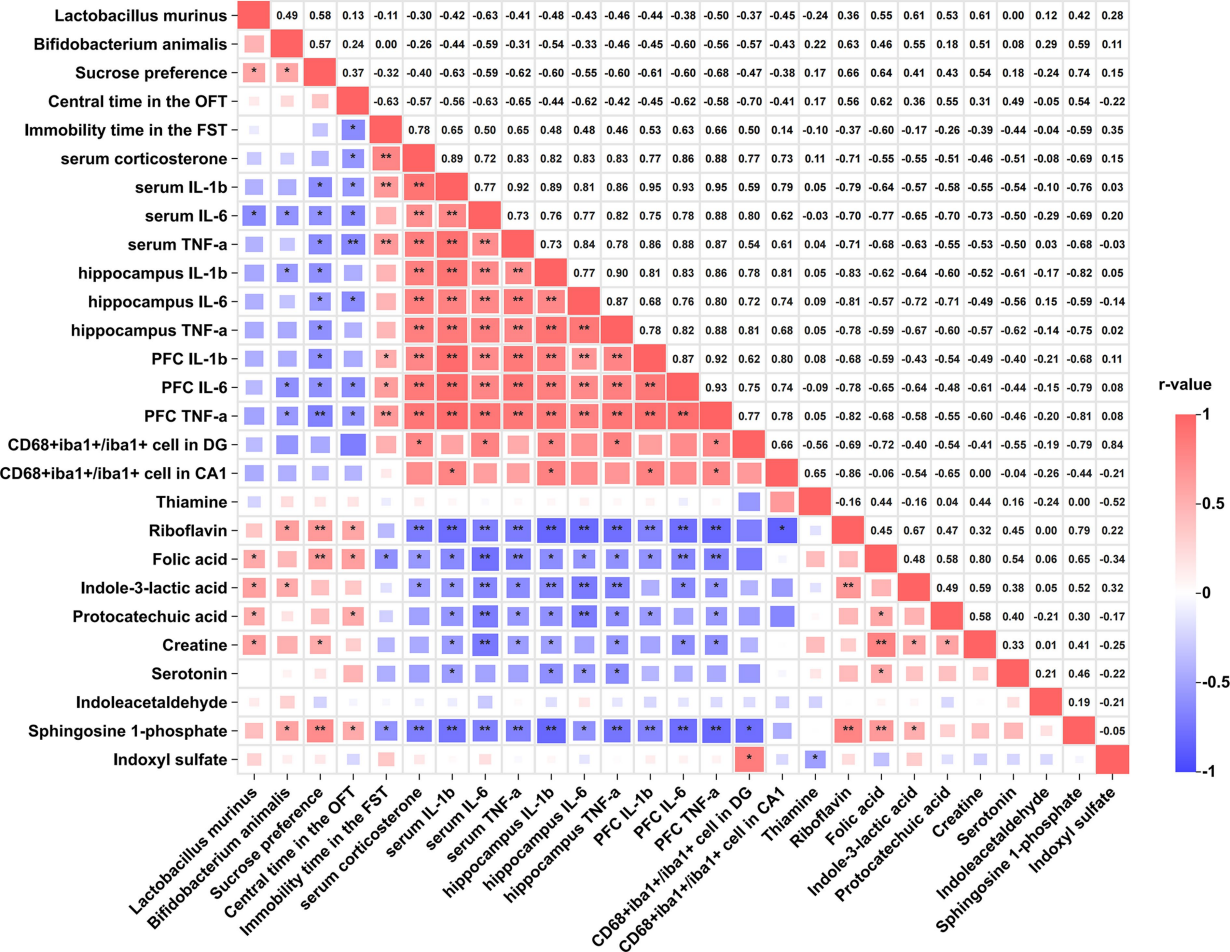
Pearson correlation analysis among depressive-like behaviors, microbial features, plasma metabolites and pro-inflammatory factors. In the upper triangle, the numbers represent the correlation coefficient r; in the lower triangle, the symbols “*” indicate significance. *p < 0.05; **p < 0.01.

To further investigate the relationship among the depressive-like behaviors, pro-inflammatory factors, and key gut microbiota at species level and plasma metabolites that showed significant changes between rats in CUMS and taVNS groups, Pearson correlation analysis was conducted. Figure 9 illustrated that *Lactobacillus murinus* and *Bifidobacterium animalis* showed significant positive correlations with the sucrose preference ratio and plasma metabolites including ILA, riboflavin, S1P, SP and creatine. Conversely, these bacteria exhibited significant negative correlations with serum IL-6, hippocampus IL-1β and PFC TNF-α. Depressive-like behaviors demonstrated significant positive correlations with pro-inflammatory factor levels and significant negative correlations with plasma concentrations of riboflavin, S1P, Sa1P and creatine. Pro-inflammatory factors showed significant negative correlations with plasma concentrations of serotonin, ILA, riboflavin, S1P, Sa1P, SP and creatine.

## 4 Discussion

**Figure 10:**
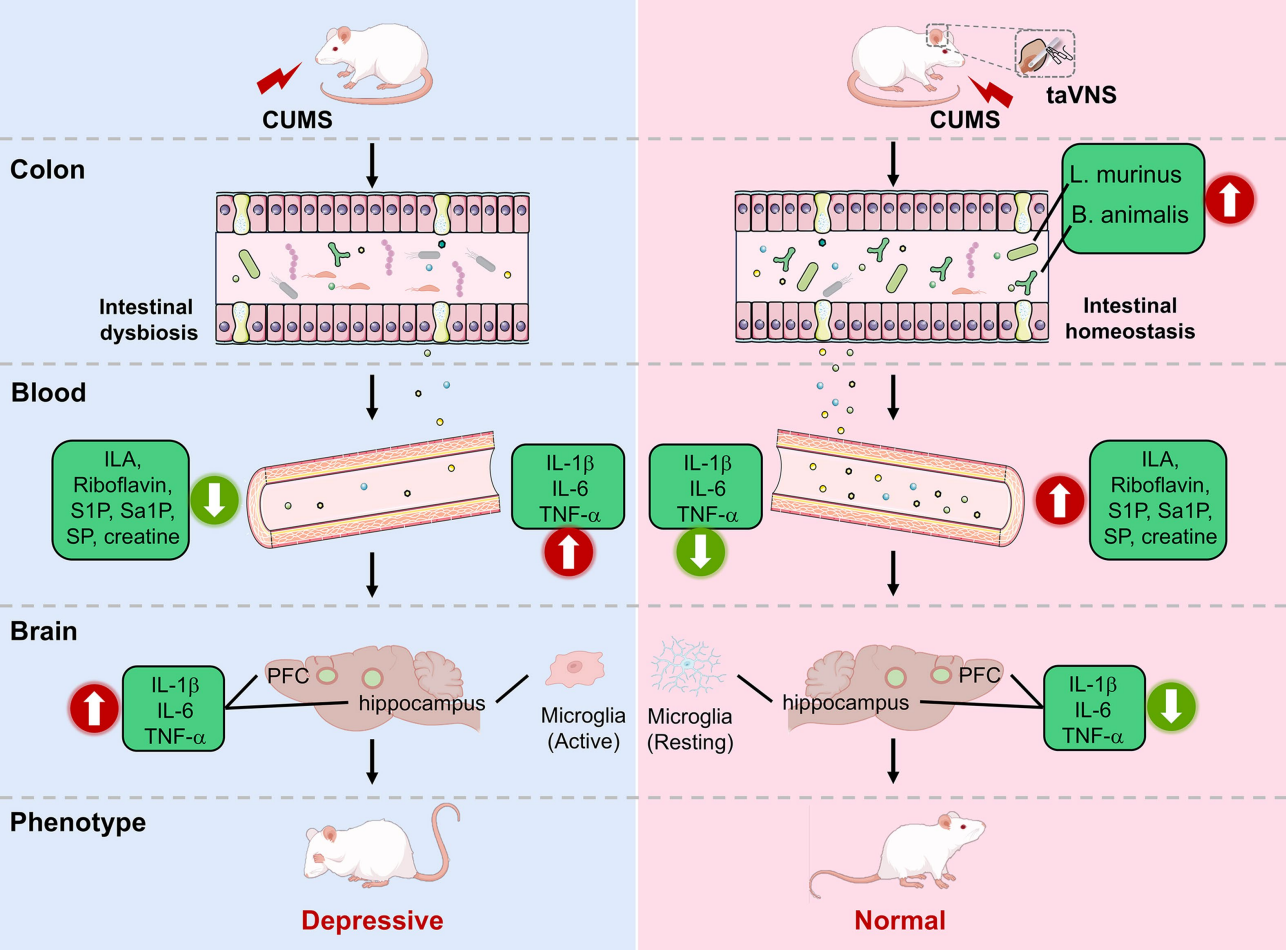
taVNS alleviates depression by enriching *Lactobacillus murinus* and *Bifidobacterium animalis* to enhance biosynthesis of microbiota-derived metabolites (ILA, riboflavin) and modulates host plasma metabolites (S1P, Sa1P, SP, creatine), thereby attenuating blood, prefrontal cortex and hippocampus inflammatory processes. Indole-3-lactic acid, ILA; Sphingosine-1-phosphate, S1P; Sphinganine-1-phosphate, Sa1P; Sphingosine, SP.

Gut microbial and metabolic dysbiosis are implicated in MDD pathogenesis, offering novel therapeutic targets (Liu et al., 2023). While taVNS efficacy in depression is established (Rong et al., 2016; Tan et al., 2023), however, whether the gut microbiome and metabolism play a role in treatment effect of taVNS on depression remains unclear. In this study, we performed integrative analysis to demonstrate the effect of taVNS on the composition of gut microbiota, plasma metabolism profile and pro-inflammatory responses in a CUMS-induced rat model of depression. Further correlation analysis indicated that taVNS may achieve the treatment effect for depression via increasing the abundance of *Lactobacillus murinus* and *Bifidobacterium animalis*, and in turn elevated the plasma concentration of riboflavin, indole-3-lactic acid and sphingosine 1-phosphate to ameliorate peripheral blood and central hippocampus and PFC inflammation (Figure 10).

### 4.1 Effects of taVNS on CUMS-induced depressive and anxiety-like behaviors

Chronic stress can lead to generalized depressive disorder in humans. As a classical model, CUMS mimics the role of naturalistic socio-environmental stressors-induced depression (Song and Kim, 2021). In this study, 4-week CUMS successfully induced depression in rats, evidenced by reduced body weight, sucrose preference, central activity, locomotion, and increased immobility. Crucially, 3-week taVNS treatment reversed all these behavioral deficits, confirming its therapeutic efficacy.

### 4.2 Effects of taVNS on GM composition and microbial functions in CUMS rats

Bidirectional signaling along the GBA axis is well-established. The brain regulates gastrointestinal function via neuroendocrine-immunological pathways and indirectly modulates microbiota through microenvironmental changes (Margolis et al., 2021), indicating neuro-modulatory influences on microbiota (Korenblik et al., 2023). Conversely, gut microbiota regulate brain function via microbial metabolites (Liu et al., 2023). Although microbial alterations in depression pathogenesis are documented—linking specific taxa, diversity shifts, symptom severity, and treatment effects (Li et al., 2021; Stevens et al., 2021), findings on characteristic microbiota and diversity vary across studies. For example, consensus is absent regarding microbial richness and diversity alterations in depression models. One study reported unchanged Shannon and Simpson metrics but diminished Chao1 and ACE values (Huang M. et al., 2023), while another indicated reduced fecal microbial diversity without altered richness in CUMS-exposed rats (Li et al., 2021). Similarly, earlier mouse research observed stable community diversity and richness estimators under CUMS (Xie et al., 2022), though a separate investigation documented declines in these measures (Zhou C. H. et al., 2023). Besides, other study exhibited reductions in the Shannon, Ace, and Chao1 indices compared under CUMS, whereas the Simpson index increased (Zhou J. et al., 2025). Critically, our study found elevated alpha diversity (Observed ASV, Ace, Chao1, Simpson) in CUMS rats, further increased by taVNS. β-Diversity confirmed distinct microbial communities between CUMS and taVNS groups, indicating taVNS modulates intestinal flora diversity.

The *Firmicutes*/*Bacteroidota* ratio is implicated in depression pathogenesis, with decreased ratios observed in MDD patients and animal models due to elevated *Bacteroidota* and reduced *Firmicutes* abundance (Jiang et al., 2015; Zheng et al., 2016). These alterations are reversible by antidepressants (Zhang Y. et al., 2021) neuromodulation therapies like repetitive transcranial magnetic stimulation (Zhou C. H. et al., 2023). Our study similarly documented reduced *Firmicutes*/*Bacteroidota* ratios in CUMS rats, which taVNS treatment effectively normalized. Specifically, taVNS reversed CUMS-induced increases in *Bacteroidales* and decreases in *Lactobacillales*, *Bifidobacteriales*, and *Clostridiales*, which align with the results previous research (Zhang et al., 2025).

Further comparative species-level analysis revealed taVNS enriched *Lactobacillus murinus*, *Bifidobacterium animalis*, *Bifidobacterium pseudolongum*, *Enterococcus faecalis* and *Prevotellaceae bacterium*, while reducing *Romboutsia ilealis*. Critically, *Lactobacillus murinus* and *Bifidobacterium animalis*, diminished in depressed rodents, could ameliorate depressive behaviors upon supplementation (Huang et al., 2022; Jia et al., 2024). Specifically, in a chronic social defeat stress model, Stress-resilient rats exhibit higher *Lactobacillus murinus* abundance, and its oral administration reduces corticosterone levels and depressive-like behaviors in stress-susceptible mice (Li et al., 2023). In comorbid constipation-depression, *Bifidobacterium animalis subsp. Lactis A6* improved Hamilton Depression Scale scores and rodent depressive-like behaviors (Wang J. et al., 2025). Notably, antidepressant efficacy often involves microbiota modulation. For example, fluoxetine increases *Lactobacillus murinus* abundance, directly contributing to its therapeutic effect (Lee et al., 2025). Similarly, Semen trigonellae (Chang W. et al., 2024) and ginsenoside Rg1 (Yu et al., 2024) exerted antidepressant effects by promoting *Lactobacillus murinus* and *Bifidobacterium animalis*. Thus, taVNS likely alleviates depression by augmenting *Lactobacillus murinus* and *Bifidobacterium animalis*.

The roles of *Bifidobacterium pseudolongum*, *Enterococcus faecalis*, and *Prevotellaceae bacterium* in depression remain controversial, with varying causal evidence. *Bifidobacterium pseudolongum* showed conflicting associations that some study linked its increase to depression (Wang G. et al., 2024) while others reported behavioral improvement after supplementation (Huang H. S. et al., 2023; Zhao J. et al., 2024). Similarly, *Enterococcus faecalis* reduction correlated with symptom relief (Takahashi et al., 2019), yet its administration alleviated depression and inflammation (Takahashi et al., 2022, 2024). *Prevotellaceae* associations are predominantly correlative: decreased in unmedicated MDD patients (Xiao et al., 2024) but increased with probiotic efficacy (Yun et al., 2024), whereas whey protein isolate reduced both depression and *Prevotellaceae UCG-001* (Xia et al., 2023). These discrepancies likely reflect divergent depression models and host–microbe interaction pathways (Hao et al., 2019). *Romboutsia* genus was suggested as a protective factor against depression onset (Wu et al., 2024) and aided stress recovery (He et al., 2024). However, evidence for its species *Romboutsia ilealis* remains insufficient. Critically, bacterial species within the same genus influence depression via distinct mechanisms, including metabolite production, immune modulation, and neurotransmitter regulation, necessitating future species-level investigations (Liu et al., 2023).

In consistent with finds of previous studies (Liu et al., 2022; Li et al., 2025), further KEGG functional annotation at the species level revealed CUMS-induced enrichment in metabolic pathways (Lipid, Amino acid, Carbohydrate, and Tryptophan metabolism), indicating gut microbiota primarily responds to stress via metabolites regulation. Furthermore, we found taVNS reversed pathways including Energy metabolism, Metabolism of cofactors and vitamins, Arginine and proline metabolism, Folate biosynthesis and Riboflavin metabolism in CUMS rats, indicating that taVNS may change gut microbiota and in turn changes the host metabolism to improve depression.

### 4.3 taVNS links gut microbiota remodeling to plasma metabolic changes beyond SCFAs

GM-derived metabolites are essential for host homeostasis, with dysbiosis promoting depression pathogenesis while interventions such as probiotics ameliorate symptoms through metabolic regulation (Ma et al., 2023). SCFAs, as a class of gut microbiota (GM)-derived metabolites, serve as crucial mediators in GBA communication (Zhou X. et al., 2025). However, reports regarding the role of SCFAs in depression remain inconsistent. Some studies have reported a significant reduction in SCFAs, particularly butyrate, and supplementation with butyrate or butyrate-producing probiotics has been shown to alleviate depressive-like behaviors (Ma et al., 2025; Śliwka et al., 2025). Conversely, other studies have found no significant changes in SCFA levels in depressed rodents, while observing increased concentrations of branched-chain fatty acids, such as isobutyric acid and isovaleric acid, in these animal models (Tanelian et al., 2023). In our study, neither CUMS nor taVNS significantly affected the majority of colonic SCFAs concentrations, but CUMS itself elevated the level of certain branched-chain fatty acids, such as isobutyric acid and isovaleric acid, and taVNS did not reverse this change. The limited response of colonic SCFAs indicated that these localized fermentation products might not be the primary drivers of the antidepressant-like effects observed in our taVNS model. Since depression is a complex disorder involving systemic and central nervous system dysfunction, the stability of local gut metabolites does not necessarily imply a lack of activity along the MGB axis (Margolis et al., 2021). Therefore, we expanded our investigation from the local gut environment to the systemic circulation via plasma metabolomics to identify circulating signals that could bridge gut dysbiosis with altered brain function. This transition is crucial as circulating metabolites are more likely than localized gut products to cross the blood–brain barrier or modulate systemic immune pathways, serving as long-range carriers of “gut-to-brain” signals within the MGB axis (Tan et al., 2022). Furthermore, plasma metabolomic profiling helps clarify how systemic neuroinflammation is regulated when localized SCFA signals are insufficient to explain the therapeutic outcome (Liu et al., 2023).

Consequently, we conducted plasma metabolomic analysis, which revealed CUMS-induced disruptions in lipid metabolism. Specifically, we observed abnormalities in glycerophospholipid, glycerolipid, α-linolenic acid, and arachidonic acid metabolism, findings that align with both previously reported depression-associated metabolic dysregulation (Tian et al., 2022) and our study’s gene function prediction results regarding GM. Crucially, taVNS modulated key pathways implicated in KEGG annotation, including Sphingolipid metabolism, One carbon pool by folate, Riboflavin metabolism, Biosynthesis of cofactors and Amino acid metabolism. These pathways upregulated by taVNS are not merely markers of general homeostasis but are deeply involved in the neuro-immunological and neuro-biochemical pathology of depression, providing a direct mechanism for the efficacy of taVNS (Tyler, 2025). Specifically, sphingolipid metabolism is critical for maintaining neuronal membrane integrity and signaling; its key metabolites, such as S1P, have been shown to inhibit the activation of the NLRP3 inflammasome in microglia, which aligns with the reduction in hippocampal neuroinflammation we observed (Werner et al., 2025). Moreover, the one-carbon pool and riboflavin metabolism are essential enzymatic cofactors for the biosynthesis of monoamine neurotransmitters, such as serotonin and dopamine, and their elevation enhances the antioxidant capacity of the hippocampus and PFC (McNulty et al., 2023).

Importantly, these improvements in plasma metabolic pathways are logically consistent with the microbial remodeling reported in our earlier sections. In this study, taVNS significantly enriched the abundance of *Lactobacillus murinus* and *Bifidobacterium animalis*, taxa that are well-documented producers of B-vitamins, one-carbon precursors, and indole derivatives (Solopova et al., 2020; Qian et al., 2024). This sequence, from increased gut microbial abundance to elevated circulating metabolites and subsequent suppression of central inflammation, illustrates a cohesive MGB axis framework that aVNS restructures the intestinal “metabolic factory” via the vagus nerve to continuously supply the circulation with neuroprotective metabolites. This synergy provides the biochemical foundation for the alleviation of depressive symptoms (McCarville et al., 2020). These findings collectively indicate that taVNS effectively ameliorates CUMS-induced depressive-like behaviors by reshaping the plasma metabolome, specifically through the generation of metabolites that harmonize with gut microbiota remodeling (Zhang et al., 2025).

### 4.4 Amelioration of blood, hippocampus and PFC inflammation by taVNS in CUMS rats

Inflammation plays a key role in the pathogenesis of depression, with elevated pro-inflammatory cytokines being a common feature in depressed patients and animal models (Yin et al., 2024). Direct administration of IL-1, TNF-α, and IFN-γ induces clinically relevant depressive symptoms, and cytokine levels typically respond to antidepressant treatment, with IL-6 decreasing and IL-22 increasing post-treatment (Więdłocha et al., 2018). Consistent with these findings, our study found significantly elevated levels of pro-inflammatory cytokines (TNF-α, IL-1β, and IL-6) in both the periphery (serum) and the central nervous system (hippocampus and PFC), accompanied by increased serum corticosterone levels. Importantly, all of these changes were reversed by taVNS treatment. Microglia, being the CNS-resident immune cells, play a critical role in regulating the neural environment. Following sustained stress, microglia can polarize toward the M1 (pro-inflammatory) phenotype (Du Preez et al., 2021), which exacerbates inflammation by expressing cytokines such as TNF-α, IL-1β, and IL-6. Growing evidence suggests that activated microglia in the hippocampus are implicated in triggering depression (Wu and Zhang, 2023). Therefore, to evaluate taVNS’s therapeutic effect specifically on depression-associated microglial activation, we performed immunofluorescence analysis. The results demonstrated that taVNS significantly reduced the number of activated microglia in the DG and CA1 subregions of the hippocampus, a finding consistent with previous reports (Wang et al., 2021).

### 4.5 Relationships among depressive-like behaviors, microbial features, plasma metabolites and pro-inflammatory factors

To elucidate taVNS mechanisms in alleviating depressive-like behaviors, we analyzed correlations between behavioral outcomes, gut microbiota, plasma metabolites, and pro-inflammatory factors. Higher sucrose preference (reduced anhedonia) positively correlated with *Lactobacillus murinus*, *Bifidobacterium animalis*, and plasma ILA, riboflavin, S1P, Sa1P, SP, and creatine. Critically, all these parameters inversely associated with pro-inflammatory factors.

Though tryptophan-serotonin dysregulation is implicated in depression (Xue et al., 2023) circulating serotonin’s clinical relevance remains contested. While some studies report reduced plasma serotonin in major depressive episodes (Colle et al., 2020), meta-analyses show no significant differences in unmedicated females (Huang et al., 2021). Consistent with this ambiguity, we observed no significant correlation between serotonin and depressive behaviors. Gut microbiota modulate depression via tryptophan metabolism into indole derivatives (Zhou Y. et al., 2023). ILA, an antidepressant indole metabolite, emerges as a convergent mediator in depression therapy. Critically, serum ILA rises in treatment-naïve MDD patients responding to antidepressants (Bhattacharyya et al., 2025), paralleling animal studies where herbal antidepressants elevate colonic ILA (Yue et al., 2024). *Lactobacillus murinus* supplementation reduces hippocampal and prefrontal cortex (PFC) neuroinflammation via ILA (Chen et al., 2024) while *Bifidobacterium breve* reverses ILA depletion in depressed mice through Aldh-dependent synthesis (Qian et al., 2024). Circulating ILA activates microglial aryl hydrocarbon receptor (AhR), suppressing IL-1β/IL-6 and corticosterone. Similar effects occur with other ILA producers like *Pediococcus acidilactici* (Wang Y. et al., 2025), and in methamphetamine withdrawal models via ILA/AhR signaling (Wang X. et al., 2025). In our study, taVNS enriched *Lactobacillus murinus* and *Bifidobacterium animalis*, with abundances positively correlating with ILA but inversely with hippocampal/PFC neuroinflammation. This suggests that the anti-depressant effect of taVNS is significantly associated with enrichment of ILA producers whose metabolite engages microglial AhR to suppress neuroinflammation, suggesting a potential mechanistic pathway.

Riboflavin (vitamin B₂), synthesized by gut microbes, modulates depression through anti-inflammatory, antioxidant, and energy metabolism pathways (McNulty et al., 2023; Śliwiński and Gawlik-Kotelnicka, 2024). Its metabolite flavin mononucleotide inhibits microglial TNFR1/NF-κB signaling (Zhang et al., 2023). Clinically, riboflavin deficiency correlates with late-life, postpartum, and post-stroke depression (Kim et al., 2018; Lin et al., 2019; Moore et al., 2019), while higher intake reduces symptom severity (Rouhani et al., 2023). Depression-alleviating interventions consistently elevate riboflavin (Luo et al., 2023; Lu et al., 2025). Our data showed riboflavin positively correlated with sucrose preference but negatively with pro-inflammatory cytokines and activated microglia. Given *Bifidobacterium* and Lactobacillus synthesize riboflavin (Averianova et al., 2020; Solopova et al., 2020), *Bifidobacterium animalis* abundance strongly linked to host riboflavin. Thus, taVNS may improve depression by boosting *Bifidobacterium animalis*-driven riboflavin production, with downstream metabolites attenuating microglia-induced neuroinflammation.

Sphingolipid metabolism, particularly S1P signaling—is central to depression pathophysiology (Yan et al., 2025). Depressed patients exhibit reduced plasma SP/S1P inversely correlating with symptom severity and inflammation (Werner et al., 2025). Symptom improvement during psychotherapy coincides with rising sphingosine/S1P, consistent with stress-induced hippocampal S1P receptor 3 (S1P3) downregulation driving depressive behaviors; its restoration rescues synaptic function via RhoA/ROCK1 (Liu H. et al., 2025). S1P3 upregulation in the medial PFC characterizes stress resilience, and blood S1P3 inversely correlates with PTSD severity (Corbett et al., 2019). Therapeutically, escitalopram and fingolimod (a sphingosine analog) normalize sphingolipid metabolism to ameliorate depression, with fingolimod suppressing NF-κB/NLRP3-mediated neuroinflammation and promoting microglial M2 polarization (Guo et al., 2020; Duan et al., 2025). Our data demonstrate that CUMS decreases plasma SP/S1P in rats, a deficit reversed by taVNS. SP/S1P levels positively correlated with *Bifidobacterium animalis* abundance but negatively with depressive behaviors and inflammation. This gut microbiota-SP-inflammation triad suggests microbiome modulation of depression via sphingolipid-mediated anti-inflammatory effects (Shan et al., 2020).

Endogenous creatine—a key energy metabolite—serves as an emerging antidepressant modulator. Like ketamine (Yue et al., 2025), creatine exerts antidepressant-like effects via activation of adenosine A1 and A2A receptors (Cunha et al., 2015). Beyond its established role in cellular energetics, creatine demonstrates dual neuro-immunological actions: suppressing pro-inflammatory macrophage polarization via JAK-STAT1/NF-κB inhibition (Ji et al., 2019; Kreider and Stout, 2021) while promoting IL-4-driven anti-inflammatory responses (Cunha et al., 2013). Clinically, creatine augments SSRI efficacy in women with MDD (Lyoo et al., 2012) and treatment-resistant adolescents, with phosphocreatine increases on magnetic resonance spectroscopy directly tracking symptom improvement (Kondo et al., 2011, 2016). Our data revealed plasma creatine positively correlated with *Lactobacillus murinus* and *Bifidobacterium animalis* but inversely with inflammation, implicating a gut microbiota-creatine-neuroinflammation axis in taVNS’s antidepressant effects.

Critically, taVNS were reported consistently enriches Lactobacillus and *Bifidobacterium* in other clinical and preclinical studies (Shi et al., 2021; Liu et al., 2024a,b). In our CUMS model, taVNS ameliorated depressive behaviors alongside increased *Lactobacillus murinus* and *Bifidobacterium animalis* abundances. Collectively, taVNS may alleviate depression by enriching these bacteria to enhance biosynthesis of microbiota-derived metabolites (ILA, riboflavin) and modulate host plasma metabolites (S1P, Sa1P, SP, creatine), thereby attenuating systemic and neuroinflammatory processes.

### 4.6 Possible mechanism of taVNS changing GM composition

Direct evidence regarding how taVNS regulates gut microbiota remains limited, however, there are several potential indirect pathways through which taVNS may alter gut microbiota composition. taVNS may reshape gut microbiota composition by modulating gastrointestinal motility and nutrient absorption. In constipation-predominant irritable bowel syndrome mouse models, taVNS has been shown to improve defecation function and demonstrated efficacy and safety in constipation-predominant irritable bowel syndrome patients (Liu et al., 2024a,b). In functional dyspepsia rats, taVNS promoted gastric motility and emptying by upregulating acetylcholine and α7 nicotinic acetylcholine receptor expression while inhibiting NF-κB p65 activation (Han et al., 2022; Rong et al., 2022). Such alterations in gastrointestinal transit speed and rhythm can directly affect the microbial colonization environment by changing intestinal content passage time, pH values, and oxygen gradients, thereby exerting selective pressure on microbial growth and metabolism (Mingyao et al., 2024). Additionally, the dorsal motor nucleus of the vagus (DMV) regulates intestinal fat absorption; its activation increases microvillus length and fat absorption, whereas inactivation produces the opposite effect (Lyu et al., 2024). This suggests that taVNS may indirectly alter the nutritional microenvironment supporting specific bacterial communities through DMV-mediated pathways.

The anti-inflammatory properties of taVNS represent another critical mechanism underlying its regulatory effects on gut microbiota. The vagus nerve suppresses systemic inflammatory responses through the cholinergic anti-inflammatory pathway (Han et al., 2025; Olovo et al., 2026). In septic patients, taVNS significantly reduces the production of pro-inflammatory cytokines such as IL-6 and TNF-α (Wu et al., 2023). Given that intestinal inflammation is a primary cause of gut dysbiosis and impaired barrier function (Olovo et al., 2026), taVNS may help restore intestinal homeostasis by attenuating inflammatory responses, thereby supporting the growth and diversity of beneficial bacteria (Han et al., 2025).

Furthermore, taVNS modulation of the central nervous system, particularly its effects on mood and stress responses, may indirectly shape gut microbiota through the gut-brain axis (Tyler, 2025). Chronic psychological stress elevates glucocorticoid levels, which drive the generation of an inflammatory subset of enteric glia that promotes monocyte- and TNF-mediated inflammation via colony-stimulating factor 1. Concurrently, glucocorticoids cause transcriptional immaturity in enteric neurons, acetylcholine deficiency, and dysmotility via transforming growth factor-β2, which further modulate gut microbiota (Schneider et al., 2023). Conversely, chronic stress suppresses central amygdala neurons, which normally activate DMV neurons to stimulate Brunner’s glands to secrete mucin that promotes Lactobacilli proliferation (Chang H. et al., 2024). Thus, taVNS may ameliorate stress-induced glucocorticoid elevation (Cuberos Paredes et al., 2025) and DMV suppression, thereby restoring mucin secretion and intestinal barrier function, ultimately exerting a positive regulatory effect on gut microbiota.

## 5 Limitations

Several limitations should be noted. Firstly, the exclusive use of male mice introduces a potential sex-related bias. The CUMS model is predominantly applied to male mice, with insufficient research on females. Secondly, while the gut microbiota might contribute to the antidepressant effects of taVNS, direct evidence is currently unavailable. Future studies will therefore investigate the direct impact of these microbial taxa on depressive-like behaviors using fecal microbiota transplantation. Thirdly, the present study did not directly assess the role of the vagus nerve in mediating the effects of taVNS. Given the critical role of the vagus nerve in the gut-brain axis, later studies should consider incorporating vagotomy experiments to elucidate the specific contribution of the vagus nerve to the antidepressant effects as well as the regulation of gut microbiome and plasm metabolism of taVNS. Lastly, neuroinflammation and alterations in neurotransmitters, are also crucial in depression. Further studies should investigate how the gut microbiota and its metabolites which were associated with the antidepressant effects of taVNS identified in this study impact neuroinflammation and brain neurotransmitters.

## 6 Conclusion

This study shows taVNS alleviates depression in rats by modulating the MGB axis. It reduced depressive behaviors and restructured gut microbiota, promoting *Bifidobacterium animalis*, and *Lactobacillus murinus* while suppressing harmful bacteria. taVNS also modulated plasma metabolism, especially metabolism of cofactor/vitamin, sphingolipid metabolism, amino and organic acid metabolism. Additionally, taVNS reduced serum, hippocampus and PFC inflammation. Furthermore, Pearson correlation analysis showed that alleviation of depressive behaviors positively correlated with *Lactobacillus murinus*, *Bifidobacterium animalis*, and plasma ILA, riboflavin, S1P, Sa1P, SP, and creatine and all these parameters inversely associated with pro-inflammatory factors. These indicates that taVNS may alleviate depression by enriching these bacteria to enhance biosynthesis of microbiota-derived metabolites (ILA, riboflavin) and modulate host plasma metabolites (S1P, Sa1P, SP, creatine), thereby attenuating systemic and neuroinflammatory processes.

## Data Availability

The data presented in the study are deposited in the NCBI Sequence Read Archive (SRA) repository, under BioProject accession number PRJNA1479870.

## Footnotes

1
https://www.chiplot.online/gene_cluster.html

## Supplementary material

SUPPLEMENTARY FIGURE S1 The Firmicutes/Bacteroidota ratio among the three groups.

## Glossary

AhR: Aryl hydrocarbon receptor
CUMS: Chronic unpredictable mild stress
DG: Dentate gyrus
DMV: Dorsal motor nucleus of the vagus
FST: Forced swim test
IL-1β: Interleukin 1 beta
IL-6: Interleukin 6
ILA: Indole-3-lactic acid
LEfSe: Linear discriminant analysis effect size
MDD: Major depressive disorder
MGB: Microbiota-gut-brain
OFT: Open-field test
OPLS-DA: Orthogonal partial least squares discriminant analysis
PCA: Principal component analysis
S1P3: S1P receptor 3
Sa1P: Sphinganine-1-phosphate
S1P: Sphingosine-1-phosphate
SP: Sphingosine
SCFAs: Short-chain fatty acids
SPT: Sucrose preference test
taVNS: Transcutaneous auricular vagus nerve stimulation
TNF-α: Tumor necrosis factor alpha

## References

[ref1] Averianova L. A. Balabanova L. A. Son O. M. Podvolotskaya A. B. Tekutyeva L. A. (2020). Production of vitamin B2 (riboflavin) by microorganisms: an overview. Front. Bioeng. Biotechnol. 8:570828. doi: 10.3389/fbioe.2020.570828, 33304888 PMC7693651

[ref2] Bhattacharyya S. MahmoudianDehkordi S. Sniatynski M. J. Belenky M. Marur V. R. Rush A. J. . (2025). Metabolomics signatures of serotonin reuptake inhibitor (escitalopram), serotonin norepinephrine reuptake inhibitor (duloxetine) and cognitive-behavioral therapy on key neurotransmitter pathways in major depressive disorder. J. Affect. Disord. 375, 397–405. doi: 10.1016/j.jad.2025.01.064, 39818336

[ref3] Chang W. Guo J. Yang Y. Zou L. Fu Y. Li M. . (2024). Semen trigonellae alleviates LPS-induced depressive behavior via enhancing the abundance of ligilactobacillus spp. Food Sci. Nutr. 12, 9414–9427. doi: 10.1002/fsn3.4475, 39619956 PMC11606864

[ref4] Chang H. Perkins M. H. Novaes L. S. Qian F. Zhang T. Neckel P. H. . (2024). Stress-sensitive neural circuits change the gut microbiome via duodenal glands. Cell 187, 5393–5412.e30. doi: 10.1016/j.cell.2024.07.019, 39121857 PMC11425084

[ref5] Chen J. Zhang C. Yang Z. Wu W. Zou W. Xin Z. . (2024). Intestinal microbiota imbalance resulted by anti-toxoplasma gondii immune responses aggravate gut and brain injury. Parasites Vectors 17:284. doi: 10.1186/s13071-024-06349-8, 38956725 PMC11221008

[ref6] Cipriani A. Furukawa T. A. Salanti G. Chaimani A. Atkinson L. Z. Ogawa Y. . (2018). Comparative efficacy and acceptability of 21 antidepressant drugs for the acute treatment of adults with major depressive disorder: a systematic review and network meta-analysis. Lancet 391, 1357–1366. doi: 10.1016/S0140-6736(17)32802-7, 29477251 PMC5889788

[ref7] Colle R. Masson P. Verstuyft C. Fève B. Werner E. Boursier-Neyret C. . (2020). Peripheral tryptophan, serotonin, kynurenine, and their metabolites in major depression: a case-control study. Psychiatry Clin. Neurosci. 74, 112–117. doi: 10.1111/pcn.12944, 31599111

[ref8] Corbett B. F. Luz S. Arner J. Pearson-Leary J. Sengupta A. Taylor D. . (2019). Sphingosine-1-phosphate receptor 3 in the medial prefrontal cortex promotes stress resilience by reducing inflammatory processes. Nat. Commun. 10:3146. doi: 10.1038/s41467-019-10904-8, 31316053 PMC6637233

[ref9] Cryan J. O’Riordan K. Cowan C. Sandhu K. Bastiaanssen T. Boehme M. . (2019). The microbiota-gut-brain axis. Physiol. Rev. 99, 1877–2013. doi: 10.1152/physrev.00018.2018 31460832

[ref10] Cuberos Paredes E. Goyes D. Mak S. Yardimian R. Ortiz N. McLaren A. . (2025). Transcutaneous auricular vagus nerve stimulation inhibits mental stress-induced cortisol release-potential implications for inflammatory conditions. Physiol. Rep. 13:e70251. doi: 10.14814/phy2.70251, 39936474 PMC11815478

[ref11] Cunha M. P. Pazini F. L. Oliveira Á. Machado D. G. Rodrigues A. L. S. (2013). Evidence for the involvement of 5-HT1A receptor in the acute antidepressant-like effect of creatine in mice. Brain Res. Bull. 95, 61–69. doi: 10.1016/j.brainresbull.2013.01.005, 23352985

[ref12] Cunha M. P. Pazini F. L. Rosa J. M. Ramos-Hryb A. B. Oliveira Á. Kaster M. P. . (2015). Creatine, similarly to ketamine, affords antidepressant-like effects in the tail suspension test via adenosine a₁ and A2A receptor activation. Purinergic Signal. 11, 215–227. doi: 10.1007/s11302-015-9446-7, 25702084 PMC4425723

[ref13] Deligiannidis K. M. Robakis T. Homitsky S. C. Ibroci E. King B. Jacob S. . (2022). Effect of transcutaneous auricular vagus nerve stimulation on major depressive disorder with peripartum onset: a multicenter, open-label, controlled proof-of-concept clinical trial (DELOS-1). J. Affect. Disord. 316, 34–41. doi: 10.1016/j.jad.2022.07.068, 35932937

[ref14] Du Preez A. Onorato D. Eiben I. Musaelyan K. Egeland M. Zunszain P. A. . (2021). Chronic stress followed by social isolation promotes depressive-like behaviour, alters microglial and astrocyte biology and reduces hippocampal neurogenesis in male mice. Brain Behav. Immun. 91, 24–47. doi: 10.1016/j.bbi.2020.07.015, 32755644

[ref15] Duan J. Sun J. Ma X. Du P. Dong P. Xue J. . (2025). Association of escitalopram-induced shifts in gut microbiota and sphingolipid metabolism with depression-like behavior in wistar-Kyoto rats. Transl. Psychiatry 15:54. doi: 10.1038/s41398-025-03277-8, 39962083 PMC11833111

[ref16] Evrensel A. Ceylan M. E. (2015). The gut-brain axis: the missing link in depression. Clin. Psychopharmacol. Neurosci. 13, 239–244. doi: 10.9758/cpn.2015.13.3.239, 26598580 PMC4662178

[ref17] Fang J. Rong P. Hong Y. Fan Y. Liu J. Wang H. . (2016). Transcutaneous vagus nerve stimulation modulates default mode network in major depressive disorder. Biol. Psychiatry 79, 266–273. doi: 10.1016/j.biopsych.2015.03.025, 25963932 PMC4838995

[ref18] Faraji N. Payami B. Ebadpour N. Gorji A. (2025). Vagus nerve stimulation and gut microbiota interactions: a novel therapeutic avenue for neuropsychiatric disorders. Neurosci. Biobehav. Rev. 169:105990. doi: 10.1016/j.neubiorev.2024.105990, 39716559

[ref19] GBD 2017 Disease and Injury Incidence and Prevalence Collaborators (2018). Global, regional, and national incidence, prevalence, and years lived with disability for 354 diseases and injuries for 195 countries and territories, 1990-2017: a systematic analysis for the global burden of disease study 2017. Lancet 392, 1789–1858. doi: 10.1016/S0140-6736(18)32279-7, 30496104 PMC6227754

[ref20] Guo Y. Gan X. Zhou H. Zhou H. Pu S. Long X. . (2020). Fingolimod suppressed the chronic unpredictable mild stress-induced depressive-like behaviors via affecting microglial and NLRP3 inflammasome activation. Life Sci. 263:118582. doi: 10.1016/j.lfs.2020.118582, 33058911

[ref21] Han R. Song Y. Liu Y. Zhuo M. Zhong M. (2025). Cholinergic reflex control of inflammation: mechanistic and translational advances in transcutaneous auricular vagus nerve stimulation across rheumatic, metabolic and postoperative disorders. Front. Immunol. 16:1702185. doi: 10.3389/fimmu.2025.1702185, 41646976 PMC12869026

[ref22] Han J. Wei W. Wang H.-C. Zhang T. Wang Y. Hou L.-W. . (2022). Transcutaneous auricular vagus nerve stimulation promotes gastric motility by up-rgulating α7nAChR and suppressing NF-κB p65 expression in duodenum in rats with functional dyspepsia. Acupunct. Res. 47, 517–524. doi: 10.13702/j.1000-0607.20220111, 35764519

[ref23] Hao Y. Ge H. Sun M. Gao Y. (2019). Selecting an appropriate animal model of depression. Int. J. Mol. Sci. 20:4827. doi: 10.3390/ijms20194827, 31569393 PMC6801385

[ref24] Hawkins B. Montgomery M. Bokota G. Santoyo M. Giron E. Eltokhi A. (2025). Gut microbiota dysbiosis at the interface of neuropsychiatric disorders and their dermatological comorbidities. Gut Microbes 17:2574934. doi: 10.1080/19490976.2025.2574934, 41277234 PMC12645903

[ref25] He H. Zhao Z. Xiao C. Li L. Liu Y.-E. Fu J. . (2024). Gut microbiome promotes mice recovery from stress-induced depression by rescuing hippocampal neurogenesis. Neurobiol. Dis. 191:106396. doi: 10.1016/j.nbd.2023.106396 38176570

[ref26] Huang T. Balasubramanian R. Yao Y. Clish C. B. Shadyab A. H. Liu B. . (2021). Associations of depression status with plasma levels of candidate lipid and amino acid metabolites: a meta-analysis of individual data from three independent samples of US postmenopausal women. Mol. Psychiatry 26, 3315–3327. doi: 10.1038/s41380-020-00870-9, 32859999 PMC7914294

[ref27] Huang M. He Y. Tian L. Yu L. Cheng Q. Li Z. . (2023). Gut microbiota-SCFAs-brain axis associated with the antidepressant activity of berberine in CUMS rats. J. Affect. Disord. 325, 141–150. doi: 10.1016/j.jad.2022.12.166, 36610597

[ref28] Huang H.-S. Lin Y.-E. Panyod S. Chen R.-A. Lin Y.-C. Chai L. M. X. . (2023). Anti-depressive-like and cognitive impairment alleviation effects of gastrodia elata blume water extract is related to gut microbiome remodeling in ApoE−/− mice exposed to unpredictable chronic mild stress. J. Ethnopharmacol. 302:115872. doi: 10.1016/j.jep.2022.115872, 36343797

[ref29] Huang L. Lv X. Ze X. Ma Z. Zhang X. He R. . (2022). Combined probiotics attenuate chronic unpredictable mild stress-induced depressive-like and anxiety-like behaviors in rats. Front. Psych. 13:990465. doi: 10.3389/fpsyt.2022.990465, 36159940 PMC9490273

[ref30] Ji L. Zhao X. Zhang B. Kang L. Song W. Zhao B. . (2019). Slc6a8-mediated creatine uptake and accumulation reprogram macrophage polarization via regulating cytokine responses. Immunity 51, 272–284.e7. doi: 10.1016/j.immuni.2019.06.007, 31399282

[ref31] Jia L. Xiao L. Fu Y. Shao Z. Jing Z. Yuan J. . (2024). Neuroprotective effects of probiotics on anxiety- and depression-like disorders in stressed mice by modulating tryptophan metabolism and the gut microbiota. Food Funct. 15, 2895–2905. doi: 10.1039/d3fo03897a, 38404190

[ref32] Jiang H. Ling Z. Zhang Y. Mao H. Ma Z. Yin Y. . (2015). Altered fecal microbiota composition in patients with major depressive disorder. Brain Behav. Immun. 48, 186–194. doi: 10.1016/j.bbi.2015.03.016, 25882912

[ref33] Kaczmarczyk M. Antosik-Wójcińska A. Dominiak M. Święcicki Ł. (2021). Use of transcutaneous auricular vagus nerve stimulation (taVNS) in the treatment of drug-resistant depression - a pilot study, presentation of five clinical cases. Psychiatr. Pol. 55, 555–564. doi: 10.12740/PP/OnlineFirst/115191, 34460881

[ref34] Kim Y. Kim M.-C. Park H.-S. Cho I.-H. Paik J. K. (2018). Association of the anxiety/depression with nutrition intake in stroke patients. Clin. Nutr. Res. 7, 11–20. doi: 10.7762/cnr.2018.7.1.11, 29423385 PMC5796919

[ref35] Kondo D. G. Forrest L. N. Shi X. Sung Y.-H. Hellem T. L. Huber R. S. . (2016). Creatine target engagement with brain bioenergetics: a dose-ranging phosphorus-31 magnetic resonance spectroscopy study of adolescent females with SSRI-resistant depression. Amino Acids 48, 1941–1954. doi: 10.1007/s00726-016-2194-3, 26907087 PMC4974294

[ref36] Kondo D. G. Sung Y.-H. Hellem T. L. Fiedler K. K. Shi X. Jeong E.-K. . (2011). Open-label adjunctive creatine for female adolescents with SSRI-resistant major depressive disorder: a 31-phosphorus magnetic resonance spectroscopy study. J. Affect. Disord. 135, 354–361. doi: 10.1016/j.jad.2011.07.010, 21831448 PMC4641570

[ref37] Korenblik V. Brouwer M. E. Korosi A. Denys D. Bockting C. L. H. Brul S. . (2023). Are neuromodulation interventions associated with changes in the gut microbiota? A systematic review. Neuropharmacology 223:109318. doi: 10.1016/j.neuropharm.2022.109318, 36334762

[ref38] Kreider R. B. Stout J. R. (2021). Creatine in health and disease. Nutrients 13:447. doi: 10.3390/nu13020447, 33572884 PMC7910963

[ref39] Lee Y.-B. Cho Y.-J. Kim J.-K. (2025). The unique role of fluoxetine in alleviating depression and anxiety by regulating gut microbiota and the expression of vagus nerve-mediated serotonin and melanocortin-4 receptors. Biomed. Pharmacother. 182:117748. doi: 10.1016/j.biopha.2024.117748, 39671722

[ref40] Li J. Jia X. Wang C. Wu C. Qin X. (2019). Altered gut metabolome contributes to depression-like behaviors in rats exposed to chronic unpredictable mild stress. Transl. Psychiatry 9:40. doi: 10.1038/s41398-019-0391-z, 30696813 PMC6351597

[ref41] Li H. Liu P. Sun T. Li Y. Wu J. Huang Y. . (2025). Dynamic alterations of depressive-like behaviors, gut microbiome, and fecal metabolome in social defeat stress mice. Transl. Psychiatry 15:115. doi: 10.1038/s41398-025-03326-2, 40169555 PMC11961705

[ref42] Li X.-J. Wang L. Wang H.-X. Zhang L. Zhang G.-L. Rong P.-J. . (2019). The effect of transcutaneous auricular vagus nerve stimulation on treatment-resistant depression monitored by resting-state fMRI and MRS: the first case report. Brain Stimul. 12, 377–379. doi: 10.1016/j.brs.2018.11.013, 30528347

[ref43] Li H. Xiang Y. Zhu Z. Wang W. Jiang Z. Zhao M. . (2021). Rifaximin-mediated gut microbiota regulation modulates the function of microglia and protects against CUMS-induced depression-like behaviors in adolescent rat. J. Neuroinflammation 18:254. doi: 10.1186/s12974-021-02303-y, 34736493 PMC8567657

[ref44] Li S. Zhang Z. Jiao Y. Jin G. Wu Y. Xu F. . (2022). An assessor-blinded, randomized comparative trial of transcutaneous auricular vagus nerve stimulation (taVNS) combined with cranial electroacupuncture vs. citalopram for depression with chronic pain. Front. Psych. 13:902450. doi: 10.3389/fpsyt.2022.902450, 35990057 PMC9386062

[ref45] Li L.-F. Zou H.-W. Song B.-L. Wang Y. Jiang Y. Li Z.-L. . (2023). Increased lactobacillus abundance contributes to stress resilience in mice exposed to chronic social defeat stress. Neuroendocrinology 113, 563–576. doi: 10.1159/000528876, 36587608

[ref46] Lin Y.-H. Chen C.-M. Su H.-M. Mu S.-C. Chang M.-L. Chu P.-Y. . (2019). Association between postpartum nutritional status and postpartum depression symptoms. Nutrients 11:1204. doi: 10.3390/nu11061204, 31141947 PMC6628029

[ref47] Liu H. Chen S. Xiang H. Xiao J. Zhao S. Zhang X. . (2025). S1PR3 in hippocampal neurons improves synaptic plasticity and decreases depressive behavior via downregulation of RhoA/ROCK1. Prog. Neuro-Psychopharmacol. Biol. Psychiatry 137:111256. doi: 10.1016/j.pnpbp.2025.111256, 39828081

[ref48] Liu J. Dai Q. Qu T. Ma J. Lv C. Wang H. . (2024a). Ameliorating effects of transcutaneous auricular vagus nerve stimulation on a mouse model of constipation-predominant irritable bowel syndrome. Neurobiol. Dis. 193:106440. doi: 10.1016/j.nbd.2024.106440, 38369213

[ref49] Liu X. Li X. Teng T. Jiang Y. Xiang Y. Fan L. . (2022). Comparative analysis of gut microbiota and fecal metabolome features among multiple depressive animal models. J. Affect. Disord. 314, 103–111. doi: 10.1016/j.jad.2022.06.088, 35780963

[ref50] Liu P. Liu Z. Wang J. Gao M. Zhang Y. Yang C. . (2024). Immunoregulatory role of the gut microbiota in inflammatory depression. Nat. Commun. 15:3003. doi: 10.1038/s41467-024-47273-w, 38589368 PMC11001948

[ref51] Liu J. Lv C. Yin M. Zhu M. Wang B. Tian J. . (2024b). Efficacy and safety of transcutaneous auricular vagus nerve stimulation in patients with constipation-predominant irritable bowel syndrome: a single-center, single-blind, randomized controlled trial. Am. J. Gastroenterol. 120, 2139–2153. doi: 10.14309/ajg.0000000000003257, 39689011 PMC12398348

[ref52] Liu C. Tang H. Liu C. Ma J. Liu G. Niu L. . (2024). Transcutaneous auricular vagus nerve stimulation for post-stroke depression: a double-blind, randomized, placebo-controlled trial. J. Affect. Disord. 354, 82–88. doi: 10.1016/j.jad.2024.03.005, 38452937

[ref53] Liu L. Wang H. Chen X. Zhang Y. Zhang H. Xie P. (2023). Gut microbiota and its metabolites in depression: from pathogenesis to treatment. EBioMedicine 90:104527. doi: 10.1016/j.ebiom.2023.104527, 36963238 PMC10051028

[ref54] Liu T. Wang Z. Li Y. Kang X. Wang X. Ren G. . (2025). Effects of transcutaneous auricular vagal nerve stimulation on chronic constipation: a multicenter, randomized controlled study. United Eur. Gastroenterol. J. 13, 1550–1559. doi: 10.1002/ueg2.70041, 40359320 PMC12529053

[ref55] Locher C. Koechlin H. Zion S. R. Werner C. Pine D. S. Kirsch I. . (2017). Efficacy and safety of selective serotonin reuptake inhibitors, serotonin-norepinephrine reuptake inhibitors, and placebo for common psychiatric disorders among children and adolescents: a systematic review and meta-analysis. JAMA Psychiatry 74, 1011–1020. doi: 10.1001/jamapsychiatry.2017.2432, 28854296 PMC5667359

[ref56] Lu R. Yang J. Fan R. Li H. Liu Y. Wang T. . (2025). Prophylactic administration of xuesaitong soft capsule ameliorating depression-like behaviors in rats after ischemic stroke by modulating metabolic disturbance. Phytomedicine 145:156997. doi: 10.1016/j.phymed.2025.156997 40570557

[ref57] Luo X. Zhou Y. Yuan S. Chen X. Zhang B. (2023). The changes in metabolomics profile induced by intermittent theta burst stimulation in major depressive disorder: an exploratory study. BMC Psychiatry 23:550. doi: 10.1186/s12888-023-05044-9, 37516823 PMC10387200

[ref58] Lyoo I. K. Yoon S. Kim T.-S. Hwang J. Kim J. E. Won W. . (2012). A randomized, double-blind placebo-controlled trial of oral creatine monohydrate augmentation for enhanced response to a selective serotonin reuptake inhibitor in women with major depressive disorder. Am. J. Psychiatry 169, 937–945. doi: 10.1176/appi.ajp.2012.12010009, 22864465 PMC4624319

[ref59] Lyu Q. Xue W. Liu R. Ma Q. Kasaragod V. B. Sun S. . (2024). A brain-to-gut signal controls intestinal fat absorption. Nature 634, 936–943. doi: 10.1038/s41586-024-07929-5, 39261733

[ref60] Ma T. Shen X. Shi X. Sakandar H. A. Quan K. Li Y. . (2023). Targeting gut microbiota and metabolism as the major probiotic mechanism - an evidence-based review. Trends Food Sci. Technol. 138, 178–198. doi: 10.1016/j.tifs.2023.06.013

[ref61] Ma X. Shi W. Wang Z. Li S. Ma R. Zhu W. . (2025). Butyric acid and valeric acid attenuate stress-induced ferroptosis and depressive-like behaviors by suppressing hippocampal neuroinflammation. J. Transl. Med. 23:974. doi: 10.1186/s12967-025-06950-0, 40898211 PMC12403447

[ref62] Malhi G. S. Mann J. J. (2018). Depression. Lancet 392, 2299–2312. doi: 10.1016/S0140-6736(18)31948-2, 30396512

[ref63] Margolis K. G. Cryan J. F. Mayer E. A. (2021). The microbiota-gut-brain axis: from motility to mood. Gastroenterology 160, 1486–1501. doi: 10.1053/j.gastro.2020.10.066, 33493503 PMC8634751

[ref64] McCarville J. L. Chen G. Y. Cuevas V. D. Troha K. Ayres J. S. (2020). Microbiota metabolites in health and disease. Annu. Rev. Immunol. 38, 147–170. doi: 10.1146/annurev-immunol-071219-125715, 32340573

[ref65] McNulty H. Pentieva K. Ward M. (2023). Causes and clinical sequelae of riboflavin deficiency. Annu. Rev. Nutr. 43, 101–122. doi: 10.1146/annurev-nutr-061121-084407, 37603429

[ref66] Mingyao W. Jiayue W. Yiran L. Shuangling O. Jinyi X. Guanting H. . (2024). Transcutaneous auricular vagus nerve stimulation for functional gastrointestinal disorders: from understanding to application. Sci. Trad. Chinese Med. 2, 276–283. doi: 10.1097/st9.0000000000000051

[ref67] Moore K. Hughes C. F. Hoey L. Ward M. Cunningham C. Molloy A. M. . (2019). B-vitamins in relation to depression in older adults over 60 years of age: the trinity ulster department of agriculture (TUDA) cohort study. J. Am. Med. Dir. Assoc. 20, 551–557.e1. doi: 10.1016/j.jamda.2018.11.031, 30692033

[ref68] Olovo C. V. Ji Y. Ocansey D. K. W. Huang X. Xu M. (2026). *Lactobacillus helveticus* R0052-derived membrane vesicles ameliorate DSS-induced inflammatory bowel disease by modulating the gut microbiota and activating the cholinergic anti-inflammatory pathway. Int. Immunopharmacol. 171:116058. doi: 10.1016/j.intimp.2025.116058, 41512794

[ref69] Qian X. Li Q. Zhu H. Chen Y. Lin G. Zhang H. . (2024). Bifidobacteria with indole-3-lactic acid-producing capacity exhibit psychobiotic potential via reducing neuroinflammation. Cell Rep. Med. 5:101798. doi: 10.1016/j.xcrm.2024.101798, 39471819 PMC11604549

[ref70] Rong P. Hou L. Yang Y. Luo X. Wang J. Zhang J. . (2022). Multi-targeting regulation of auricular vagus nerve stimulation on gastric hypersensitivity and gastric dysmotility in a rodent model of functional dyspepsia. Gut Microb. Integr. Wellness 1, 1–10. doi: 10.54844/gmiw.2022.0088

[ref71] Rong P. Liu J. Wang L. Liu R. Fang J. Zhao J. . (2016). Effect of transcutaneous auricular vagus nerve stimulation on major depressive disorder: a nonrandomized controlled pilot study. J. Affect. Disord. 195, 172–179. doi: 10.1016/j.jad.2016.02.031, 26896810 PMC4828906

[ref72] Rouhani P. Amoushahi M. Keshteli A. H. Saneei P. Afshar H. Esmaillzadeh A. . (2023). Dietary riboflavin intake in relation to psychological disorders in iranian adults: an observational study. Sci. Rep. 13:5152. doi: 10.1038/s41598-023-32309-w, 36991113 PMC10060244

[ref73] Schneider K. M. Blank N. Alvarez Y. Thum K. Lundgren P. Litichevskiy L. . (2023). The enteric nervous system relays psychological stress to intestinal inflammation. Cell 186, 2823–2838.e20. doi: 10.1016/j.cell.2023.05.001, 37236193 PMC10330875

[ref74] Shan B. Ai Z. Zeng S. Song Y. Song J. Zeng Q. . (2020). Gut microbiome-derived lactate promotes to anxiety-like behaviors through GPR81 receptor-mediated lipid metabolism pathway. Psychoneuroendocrinology 117:104699. doi: 10.1016/j.psyneuen.2020.104699, 32402927

[ref75] Shi X. Hu Y. Zhang B. Li W. Chen J. Liu F. (2021). Ameliorating effects and mechanisms of transcutaneous auricular vagal nerve stimulation on abdominal pain and constipation. JCI Insight 6:e150052. doi: 10.1172/jci.insight.150052, 34138761 PMC8410029

[ref76] Śliwiński W. Gawlik-Kotelnicka O. (2024). Circulating B vitamins metabolites in depressive disorders - connections with the microbiota-gut-brain axis. Behav. Brain Res. 472:115145. doi: 10.1016/j.bbr.2024.115145, 38992845

[ref77] Śliwka A. Polak-Berecka M. Zdybel K. Zelek-Molik A. Waśko A. (2025). Psychobiotics in depression: sources, metabolites, and treatment-a systematic review. Nutrients 17:2139. doi: 10.3390/nu17132139, 40647242 PMC12252283

[ref78] Solopova A. Bottacini F. Venturi degli Esposti E. Amaretti A. Raimondi S. Rossi M. . (2020). Riboflavin biosynthesis and overproduction by a derivative of the human gut commensal *Bifidobacterium longum* subsp. *infantis* ATCC 15697. Front. Microbiol. 11:573335. doi: 10.3389/fmicb.2020.573335, 33042083 PMC7522473

[ref79] Song J. Kim Y.-K. (2021). Animal models for the study of depressive disorder. CNS Neurosci. Ther. 27, 633–642. doi: 10.1111/cns.13622, 33650178 PMC8111503

[ref80] Stevens B. R. Roesch L. Thiago P. Russell J. T. Pepine C. J. Holbert R. C. . (2021). Depression phenotype identified by using single nucleotide exact amplicon sequence variants of the human gut microbiome. Mol. Psychiatry 26, 4277–4287. doi: 10.1038/s41380-020-0652-5, 31988436 PMC11549940

[ref81] Takahashi K. Kurokawa K. Hong L. Miyagawa K. Mochida-Saito A. Iwasa M. . (2022). Antidepressant effects of enterococcus faecalis 2001 through the regulation of prefrontal cortical myelination via the enhancement of CREB/BDNF and NF-κB p65/LIF/STAT3 pathways in olfactory bulbectomized mice. J. Psychiatr. Res. 148, 137–148. doi: 10.1016/j.jpsychires.2022.01.047, 35123326

[ref82] Takahashi K. Nakagawasai O. Nemoto W. Odaira T. Sakuma W. Onogi H. . (2019). Effect of enterococcus faecalis 2001 on colitis and depressive-like behavior in dextran sulfate sodium-treated mice: involvement of the brain-gut axis. J. Neuroinflammation 16:201. doi: 10.1186/s12974-019-1580-7, 31672153 PMC6822456

[ref83] Takahashi K. Tsuji M. Nakagawasai O. Miyagawa K. Kurokawa K. Mochida-Saito A. . (2024). Anxiolytic effects of enterococcus faecalis 2001 on a mouse model of colitis. Sci. Rep. 14:11519. doi: 10.1038/s41598-024-62309-3, 38769131 PMC11106339

[ref84] Tan C. Qiao M. Ma Y. Luo Y. Fang J. Yang Y. (2023). The efficacy and safety of transcutaneous auricular vagus nerve stimulation in the treatment of depressive disorder: a systematic review and meta-analysis of randomized controlled trials. J. Affect. Disord. 337, 37–49. doi: 10.1016/j.jad.2023.05.048, 37230264

[ref85] Tan C. Yan Q. Ma Y. Fang J. Yang Y. (2022). Recognizing the role of the vagus nerve in depression from microbiota-gut brain axis. Front. Neurol. 13:1015175. doi: 10.3389/fneur.2022.1015175, 36438957 PMC9685564

[ref86] Tanelian A. Nankova B. Cheriyan A. Arens C. Hu F. Sabban E. L. (2023). Differences in gut microbiota associated with stress resilience and susceptibility to single prolonged stress in female rodents. Neurobiol. Stress 24:100533. doi: 10.1016/j.ynstr.2023.100533, 36970450 PMC10034505

[ref87] Tian T. Mao Q. Xie J. Wang Y. Shao W.-H. Zhong Q. . (2022). Multi-omics data reveals the disturbance of glycerophospholipid metabolism caused by disordered gut microbiota in depressed mice. J. Adv. Res. 39, 135–145. doi: 10.1016/j.jare.2021.10.002, 35777903 PMC9263645

[ref88] Tyler W. J. (2025). Transcutaneous auricular vagus nerve stimulation for treating emotional dysregulation and inflammation in common neuropsychiatric disorders. Brain Sci. 16:8. doi: 10.3390/brainsci16010008, 41594729 PMC12838753

[ref89] Vos T. Lim S. S. Abbafati C. Abbas K. M. Abbasi M. Abbasifard M. . (2020). Global burden of 369 diseases and injuries in 204 countries and territories, 1990–2019: a systematic analysis for the global burden of disease study 2019. Lancet 396, 1204–1222. doi: 10.1016/S0140-6736(20)30925-9, 33069326 PMC7567026

[ref90] Wang G. Cao L. Li S. Zhang M. Li Y. Duan J. . (2024). Gut microbiota dysbiosis-mediated ceramides elevation contributes to corticosterone-induced depression by impairing mitochondrial function. NPJ Biofilms Microbiomes 10:111. doi: 10.1038/s41522-024-00582-w, 39468065 PMC11519513

[ref91] Wang Y. Gong J. Geng K. Chen X. Jia M. Yang C. . (2025). *Pediococcus acidilactici* attenuates chronic stress-induced depression via generating metabolite indole-3-lactic acid and downregulating neuroinflammation. J. Neuroinflammation 22:267. doi: 10.1186/s12974-025-03580-7, 41239394 PMC12619292

[ref92] Wang X. Hu M. Wu W. Lou X. Gao R. Ma T. . (2025). Indole derivatives ameliorated the methamphetamine-induced depression and anxiety via aryl hydrocarbon receptor along “microbiota-brain” axis. Gut Microbes 17:2470386. doi: 10.1080/19490976.2025.2470386, 39996473 PMC11864316

[ref93] Wang J. Ren Y. Chen J. Chen S. Li X. Chen J. . (2025). *Bifidobacterium animalis* subsp. *lactis* A6 alleviates comorbid constipation and depression by rebalancing tryptophan metabolism. Sci. Bull. 70, 3738–3742. doi: 10.1016/j.scib.2025.04.042 40328604

[ref94] Wang Y. Tan Q. Pan M. Yu J. Wu S. Tu W. . (2024). Minimally invasive vagus nerve stimulation modulates mast cell degranulation via the microbiota-gut-brain axis to ameliorate blood-brain barrier and intestinal barrier damage following ischemic stroke. Int. Immunopharmacol. 132:112030. doi: 10.1016/j.intimp.2024.112030, 38603861

[ref95] Wang J.-Y. Zhang Y. Chen Y. Wang Y. Li S.-Y. Wang Y.-F. . (2021). Mechanisms underlying antidepressant effect of transcutaneous auricular vagus nerve stimulation on CUMS model rats based on hippocampal α7nAchR/NF-κB signal pathway. J. Neuroinflammation 18:291. doi: 10.1186/s12974-021-02341-6, 34920740 PMC8680337

[ref96] Werner F. Schumacher F. Mühle C. Adler W. Schug C. Schäflein E. . (2025). Psychosomatic - psychotherapeutic treatment of stress-related disorders impacts the sphingolipid metabolism towards increased sphingosine and sphingosine-1-phosphate levels. Eur. Arch. Psychiatry Clin. Neurosci. 275, 2049–2058. doi: 10.1007/s00406-025-01985-2, 40042667 PMC12589259

[ref97] Więdłocha M. Marcinowicz P. Krupa R. Janoska-Jaździk M. Janus M. Dębowska W. . (2018). Effect of antidepressant treatment on peripheral inflammation markers – a meta-analysis. Prog. Neuro-Psychopharmacol. Biol. Psychiatry 80, 217–226. doi: 10.1016/j.pnpbp.2017.04.026, 28445690

[ref98] Wu J. Ou G. Wang S. Chen Y. Xu L. Deng L. . (2024). The predictive, preventive, and personalized medicine of depression: gut microbiota and inflammation. EPMA J. 15, 587–598. doi: 10.1007/s13167-024-00379-z, 39635025 PMC11612071

[ref99] Wu A. Zhang J. (2023). Neuroinflammation, memory, and depression: new approaches to hippocampal neurogenesis. J. Neuroinflammation 20:283. doi: 10.1186/s12974-023-02964-x, 38012702 PMC10683283

[ref100] Wu Z. Zhang X. Cai T. Li Y. Guo X. Zhao X. . (2023). Transcutaneous auricular vagus nerve stimulation reduces cytokine production in sepsis: an open double-blind, sham-controlled, pilot study. Brain Stimul. 16, 507–514. doi: 10.1016/j.brs.2023.02.008, 36801260

[ref101] Xia S. Maitiniyazi G. Liu Y. Chen Y. Guo M. He J. . (2023). Whey protein isolate attenuates depression-like behavior developed in a mouse model of breast tumor. Food Res. Int. 169:112849. doi: 10.1016/j.foodres.2023.112849 37254425

[ref102] Xiao S. Yang Z. Yan H. Chen G. Zhong S. Chen P. . (2024). Gut proinflammatory bacteria is associated with abnormal functional connectivity of hippocampus in unmedicated patients with major depressive disorder. Transl. Psychiatry 14:292. doi: 10.1038/s41398-024-03012-9, 39013880 PMC11253007

[ref103] Xie Y. Wu Z. Zhou L. Sun L. Xiao L. Wang G. (2022). Swimming exercise modulates gut microbiota in CUMS-induced depressed mice. Neuropsychiatr. Dis. Treat. 18, 749–760. doi: 10.2147/NDT.S355723, 35411144 PMC8994653

[ref104] Xue C. Li G. Zheng Q. Gu X. Shi Q. Su Y. . (2023). Tryptophan metabolism in health and disease. Cell Metab. 35, 1304–1326. doi: 10.1016/j.cmet.2023.06.004, 37352864

[ref105] Yan Y. Zhang Y. Liu M. Li L. Zheng Y. (2025). Neuroprotection vs. neurotoxicity: the dual impact of brain lipids in depression. Int. J. Mol. Sci. 26:2722. doi: 10.3390/ijms26062722, 40141364 PMC11943007

[ref106] Yang J. Zheng P. Li Y. Wu J. Tan X. Zhou J. . (2020). Landscapes of bacterial and metabolic signatures and their interaction in major depressive disorders. Sci. Adv. 6:eaba8555. doi: 10.1126/sciadv.aba8555, 33268363 PMC7710361

[ref107] Yin Y. Ju T. Zeng D. Duan F. Zhu Y. Liu J. . (2024). “Inflamed” depression: a review of the interactions between depression and inflammation and current anti-inflammatory strategies for depression. Pharmacol. Res. 207:107322. doi: 10.1016/j.phrs.2024.107322, 39038630

[ref108] Yu S. Yin Z. Ling M. Chen Z. Zhang Y. Pan Y. . (2024). Ginsenoside Rg1 enriches gut microbial indole-3-acetic acid to alleviate depression-like behavior in mice via oxytocin signaling. Phytomedicine 135:156186. doi: 10.1016/j.phymed.2024.156186 39515104

[ref109] Yue Y. Ke Y. Zheng J. Wang Z. Liu H. Liu S. (2024). Microbiota-derived tryptophan metabolism and AMPK/mTOR pathway mediate antidepressant-like effect of shugan hewei decoction. Front. Pharmacol. 15:1466336. doi: 10.3389/fphar.2024.1466336, 39351096 PMC11439769

[ref110] Yue C. Wang N. Zhai H. Yuan Z. Cui Y. Quan J. . (2025). Adenosine signalling drives antidepressant actions of ketamine and ECT. Nature 649, 423–431. doi: 10.1038/s41586-025-09755-9, 41193806 PMC12779573

[ref111] Yun S.-W. Shin Y.-J. Ma X. Kim D.-H. (2024). Lactobacillus plantarum and *bifidobacterium longum* alleviate high-fat diet-induced obesity and depression/cognitive impairment-like behavior in mice by upregulating AMPK activation and downregulating adipogenesis and gut dysbiosis. Nutrients 16:3810. doi: 10.3390/nu16223810, 39599597 PMC11597813

[ref112] Zhang Y. Anoopkumar-Dukie S. Davey A. K. (2021). SIRT1 and SIRT2 modulators: potential anti-inflammatory treatment for depression? Biomolecules 11:353. doi: 10.3390/biom11030353, 33669121 PMC7996578

[ref113] Zhang M. Chen H. Zhang W. Liu Y. Ding L. Gong J. . (2023). Biomimetic remodeling of microglial riboflavin metabolism ameliorates cognitive impairment by modulating neuroinflammation. Adv. Sci. 10:e2300180. doi: 10.1002/advs.202300180, 36799538 PMC10131853

[ref114] Zhang X. He X. Zhao Y. Zou Q. Liang Y. Zhang J. . (2025). Transcutaneous auricular vagus nerve stimulation regulates gut microbiota mediated peripheral inflammation and metabolic disorders to suppress depressive-like behaviors in CUMS rats. Front. Microbiol. 16:1576686. doi: 10.3389/fmicb.2025.1576686, 40809052 PMC12344421

[ref115] Zhang W. Qu W. Wang H. Yan H. (2021). Antidepressants fluoxetine and amitriptyline induce alterations in intestinal microbiota and gut microbiome function in rats exposed to chronic unpredictable mild stress. Transl. Psychiatry 11:131. doi: 10.1038/s41398-021-01254-5, 33602895 PMC7892574

[ref116] Zhao M. Ren Z. Zhao A. Tang Y. Kuang J. Li M. . (2024). Gut bacteria-driven homovanillic acid alleviates depression by modulating synaptic integrity. Cell Metab. 36, 1000–1012.e6. doi: 10.1016/j.cmet.2024.03.010, 38582087

[ref117] Zhao J. Yuan J. Zhang Y. Deng L. Pan Y. Bai X. . (2024). Bifidobacterium pseudonumeratum W112 alleviated depressive and liver injury symptoms induced by chronic unpredictable mild stress via gut-liver-brain axis. Front. Nutr. 11:1421007. doi: 10.3389/fnut.2024.1421007, 39224184 PMC11366711

[ref118] Zheng P. Zeng B. Zhou C. Liu M. Fang Z. Xu X. . (2016). Gut microbiome remodeling induces depressive-like behaviors through a pathway mediated by the host’s metabolism. Mol. Psychiatry 21, 786–796. doi: 10.1038/mp.2016.44, 27067014

[ref119] Zhou Y. Chen Y. He H. Peng M. Zeng M. Sun H. (2023). The role of the indoles in microbiota-gut-brain axis and potential therapeutic targets: a focus on human neurological and neuropsychiatric diseases. Neuropharmacology 239:109690. doi: 10.1016/j.neuropharm.2023.109690 37619773

[ref120] Zhou C.-H. Chen Y.-H. Xue S.-S. Shi Q.-Q. Guo L. Yu H. . (2023). rTMS ameliorates depressive-like behaviors and regulates the gut microbiome and medium- and long-chain fatty acids in mice exposed to chronic unpredictable mild stress. CNS Neurosci. Ther. 29, 3549–3566. doi: 10.1111/cns.14287, 37269082 PMC10580350

[ref121] Zhou X. Wang S. Wang X. Chen X. Zhou P. Ma K. . (2025). Mechanisms of the effect of gut microbes on depression through the microbiota-gut-brain axis. Front. Nutr. 12:1634548. doi: 10.3389/fnut.2025.1634548, 40843192 PMC12364656

[ref122] Zhou J. Zhao Y. Li Y. Li J. Huang J. Liu L. . (2025). Jasmine tea extract prevents CUMS-induced depression-like behaviors through the modulation of microbiota-gut-brain axis. Food Res. Int. 209:116214. doi: 10.1016/j.foodres.2025.116214, 40253129

[ref123] Zou N. Zhou Q. Qin Z. Ma L. Wang H. Su L. . (2025). Transcutaneous auricular vagus nerve stimulation for acquired immune deficiency syndrome patients with depressive symptoms: a pilot randomized clinical trial. Brain Stimul. 18, 987–989. doi: 10.1016/j.brs.2025.04.022, 40316249

[ref124] Zyr X. Jj F. Xq F. Ll X. Gf J. Mh L. . (2025). Effectiveness and safety of transcutaneous auricular vagus nerve stimulation for depression in patients with epilepsy. Epilepsy Behav. 163:110226. doi: 10.1016/j.yebeh.2024.110226 39675145

