# Supplementary figures and images for: Transcutaneous auricular vagus nerve stimulation improves depressive-like behaviors in CUMS rats through regulation of gut microbiome, serum metabolites, and immune factors
Source: Front Microbiol. 2026 Jul 1;17:1820578. doi: 10.3389/fmicb.2026.1820578 (PMC13369481; doi:10.3389/fmicb.2026.1820578)

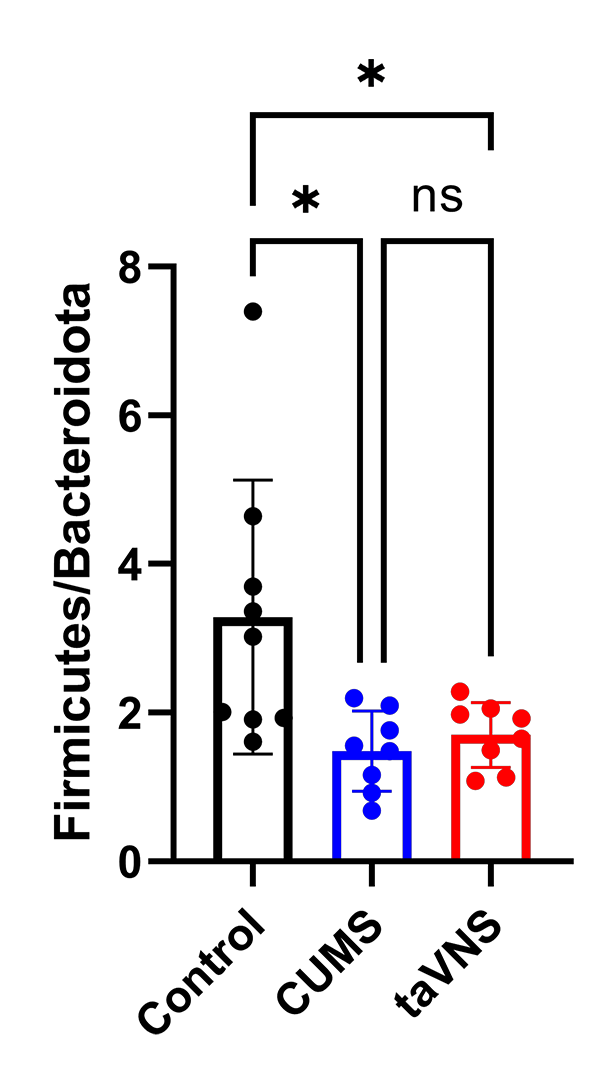

Supplement: SUPPLEMENTARY FIGURE S1 — The Firmicutes/Bacteroidota ratio among the three groups. [file Image_1.tif]
