# Supplementary material for: Transcutaneous auricular vagus nerve stimulation improves depressive-like behaviors in CUMS rats through regulation of gut microbiome, serum metabolites, and immune factors
Source: Front Microbiol. 2026 Jul 1;17:1820578. doi: 10.3389/fmicb.2026.1820578 (PMC13369481; doi:10.3389/fmicb.2026.1820578)
Supplement: Supplementary file 2 [file Table_1.DOCX]

| **Taxonomy** | **mean(taVNS group)** | **variance(taVNS group)** | **stderr(taVNS group)** | **mean(CUMS group)** | **variance(CUMS group)** | **stderr(CUMS group)** | **p.value** | **q.value** |
| --- | --- | --- | --- | --- | --- | --- | --- | --- |
| o__Eubacteriales | 0 | 0 | 0 | 0.000044 | 0 | 0.000022 | 0.000999 | 0.023976 |
| o__Rhizobiales | 0.000033 | 0 | 0.000033 | 0 | 0 | 0 | 0.000999 | 0.023976 |
| o__Bifidobacteriales | 0.000946 | 0.000002 | 0.000486 | 0.009164 | 0.000142 | 0.004216 | 0.021978 | 0.351648 |
| o__Micrococcales | 0.0006 | 0 | 0.000209 | 0.000121 | 0 | 0.000083 | 0.04995 | 0.5466528 |

**Table S1. Metastats analysis of gut micobiome between CUMS and taVNS groups at order level.**
