# Supplementary material for: Transcutaneous auricular vagus nerve stimulation improves depressive-like behaviors in CUMS rats through regulation of gut microbiome, serum metabolites, and immune factors
Source: Front Microbiol. 2026 Jul 1;17:1820578. doi: 10.3389/fmicb.2026.1820578 (PMC13369481; doi:10.3389/fmicb.2026.1820578)
Supplement: Supplementary file 3 [file Table_2.DOCX]

**Table S2. Metastats analysis of gut micobiome between CUMS and taVNS groups at genera level.**

| **Taxonomy** | **mean(taVNS group)** | **variance(taVNS group)** | **stderr(taVNS group)** | **mean(CUMS group)** | **variance(CUMS group)** | **stderr(CUMS group)** | **p.value** | **q.value** |
| --- | --- | --- | --- | --- | --- | --- | --- | --- |
| g__Methanobrevibacter | 0.005147 | 0.000119 | 0.003865 | 0 | 0 | 0 | 0.000999 | 0.049617 |
| g__Streptococcus | 0.001057 | 0.000001 | 0.000259 | 0.000055 | 0 | 0.000022 | 0.000999 | 0.049617 |
| g__Eubacterium | 0 | 0 | 0 | 0.000044 | 0 | 0.000022 | 0.000999 | 0.049617 |
| g__Rodentibacter | 0.00014 | 0 | 0.000037 | 0.000018 | 0 | 0.000012 | 0.001998 | 0.0744255 |
| g__Bifidobacterium | 0.000946 | 0.000002 | 0.000486 | 0.009164 | 0.000142 | 0.004216 | 0.021978 | 0.5209785 |
| g__Eisenbergiella | 0.000007 | 0 | 0.000007 | 0.00025 | 0 | 0.000142 | 0.021978 | 0.5209785 |
| g__Oxalobacter | 0.000007 | 0 | 0.000007 | 0.000063 | 0 | 0.000039 | 0.02997 | 0.5209785 |
| g__Rothia | 0.000118 | 0 | 0.000049 | 0.000007 | 0 | 0.000007 | 0.031968 | 0.5209785 |
| g__Ligilactobacillus | 0.044673 | 0.000882 | 0.010498 | 0.102597 | 0.004506 | 0.023733 | 0.032967 | 0.5209785 |
| g__Leucobacter | 0.000044 | 0 | 0.000025 | 0 | 0 | 0 | 0.034965 | 0.5209785 |
| g__Monoglobus | 0.007728 | 0.000018 | 0.001497 | 0.004065 | 0.000004 | 0.00074 | 0.047952 | 0.649531636 |
