# Supplementary material for: Transcutaneous auricular vagus nerve stimulation improves depressive-like behaviors in CUMS rats through regulation of gut microbiome, serum metabolites, and immune factors
Source: Front Microbiol. 2026 Jul 1;17:1820578. doi: 10.3389/fmicb.2026.1820578 (PMC13369481; doi:10.3389/fmicb.2026.1820578)
Supplement: Supplementary file 4 [file Table_3.DOCX]

**Table S3. Tax4Fun2 analysis of gut microbiome among Control, CUMS and taVNS groups at level 2.**

| Pathway | Group | | Mean Deviation | Standard error | P-value | 95% Confidence interval | |
| --- | --- | --- | --- | --- | --- | --- | --- |
|  |  |  |  |  |  | Lower-bound | Upper-bound |
| Amino acid metabolism | Control | CUMS | -.00897551844^*^ | 0.0019 | 0.0000 | -0.0129 | -0.0050 |
|  |  | taVNS | -.00812064219^*^ | 0.0019 | 0.0000 | -0.0121 | -0.0042 |
|  | CUMS | Control | .00897551844^*^ | 0.0019 | 0.0000 | 0.0050 | 0.0129 |
|  |  | taVNS | 0.0009 | 0.0020 | 0.6690 | -0.0032 | 0.0049 |
|  | taVNS | Control | .00812064219^*^ | 0.0019 | 0.0000 | 0.0042 | 0.0121 |
|  |  | CUMS | -0.0009 | 0.0020 | 0.6690 | -0.0049 | 0.0032 |
| Carbohydrate metabolism | Control | CUMS | .01882661750^*^ | 0.0032 | 0.0000 | 0.0122 | 0.0254 |
|  |  | taVNS | .01201298075^*^ | 0.0032 | 0.0010 | 0.0054 | 0.0186 |
|  | CUMS | Control | -.01882661750^*^ | 0.0032 | 0.0000 | -0.0254 | -0.0122 |
|  |  | taVNS | -.00681363675^*^ | 0.0033 | 0.0500 | -0.0136 | 0.0000 |
|  | taVNS | Control | -.01201298075^*^ | 0.0032 | 0.0010 | -0.0186 | -0.0054 |
|  |  | CUMS | .00681363675^*^ | 0.0033 | 0.0500 | 0.0000 | 0.0136 |
| Energy metabolism | Control | CUMS | -.00239826126^*^ | 0.0007 | 0.0020 | -0.0038 | -0.0010 |
|  |  | taVNS | 0.0002 | 0.0007 | 0.7320 | -0.0012 | 0.0017 |
|  | CUMS | Control | .00239826126^*^ | 0.0007 | 0.0020 | 0.0010 | 0.0038 |
|  |  | taVNS | .00263464613^*^ | 0.0007 | 0.0010 | 0.0012 | 0.0041 |
|  | taVNS | Control | -0.0002 | 0.0007 | 0.7320 | -0.0017 | 0.0012 |
|  |  | CUMS | -.00263464613^*^ | 0.0007 | 0.0010 | -0.0041 | -0.0012 |
| Lipid metabolism | Control | CUMS | -.00166948803^*^ | 0.0006 | 0.0140 | -0.0030 | -0.0004 |
|  |  | taVNS | -.00173118503^*^ | 0.0006 | 0.0110 | -0.0030 | -0.0004 |
|  | CUMS | Control | .00166948803^*^ | 0.0006 | 0.0140 | 0.0004 | 0.0030 |
|  |  | taVNS | -0.0001 | 0.0006 | 0.9240 | -0.0014 | 0.0013 |
|  | taVNS | Control | .00173118503^*^ | 0.0006 | 0.0110 | 0.0004 | 0.0030 |
|  |  | CUMS | 0.0001 | 0.0006 | 0.9240 | -0.0013 | 0.0014 |
| Metabolism of cofactors and vitamins | Control | CUMS | -.00206347786^*^ | 0.0008 | 0.0150 | -0.0037 | -0.0004 |
|  |  | taVNS | 0.0001 | 0.0008 | 0.9440 | -0.0016 | 0.0017 |
|  | CUMS | Control | .00206347786^*^ | 0.0008 | 0.0150 | 0.0004 | 0.0037 |
|  |  | taVNS | .00211960188^*^ | 0.0008 | 0.0150 | 0.0004 | 0.0038 |
|  | taVNS | Control | -0.0001 | 0.0008 | 0.9440 | -0.0017 | 0.0016 |
|  |  | CUMS | -.00211960188^*^ | 0.0008 | 0.0150 | -0.0038 | -0.0004 |
| Metabolism of other amino acids | Control | CUMS | 0.0001 | 0.0002 | 0.3570 | -0.0002 | 0.0005 |
|  |  | taVNS | 0.0002 | 0.0002 | 0.1600 | -0.0001 | 0.0006 |
|  | CUMS | Control | -0.0001 | 0.0 002 | 0.3570 | -0.0005 | 0.0002 |
|  |  | taVNS | 0.0001 | 0.0002 | 0.6210 | -0.0003 | 0.0004 |
|  | taVNS | Control | -0.0002 | 0.0002 | 0.1600 | -0.0006 | 0.0001 |
|  |  | CUMS | -0.0001 | 0.0002 | 0.6210 | -0.0004 | 0.0003 |
