# Supplementary material for: Transcutaneous auricular vagus nerve stimulation improves depressive-like behaviors in CUMS rats through regulation of gut microbiome, serum metabolites, and immune factors
Source: Front Microbiol. 2026 Jul 1;17:1820578. doi: 10.3389/fmicb.2026.1820578 (PMC13369481; doi:10.3389/fmicb.2026.1820578)
Supplement: Supplementary file 5 [file Table_4.DOCX]

**Table S4. Tax4Fun2 analysis of gut microbiome among Control, CUMS and taVNS groups at level 3.**

| Pathway | Group | | Mean Deviation | Standard error | P-value | 95% Confidence interval | |
| --- | --- | --- | --- | --- | --- | --- | --- |
|  |  |  |  |  |  | Lower-bound | Upper-bound |
| Arginine and proline metabolism | Control | CUMS | -.00073965061^*^ | 0.000236211 | 0.005 | -0.001229523 | -0.000249778 |
|  |  | taVNS | -0.000209457 | 0.000236211 | 0.385 | -0.000699329 | 0.000280416 |
|  | CUMS | Control | .00073965061^*^ | 0.000236211 | 0.005 | 0.000249778 | 0.001229523 |
|  |  | taVNS | .00053019375^*^ | 0.00024306 | 0.04 | 0.000026119 | 0.001034269 |
|  | taVNS | Control | 0.000209457 | 0.000236211 | 0.385 | -0.000280416 | 0.000699329 |
|  |  | CUMS | -.00053019375^*^ | 0.00024306 | 0.04 | -0.001034269 | -0.000026119 |
| Folate biosynthesis | Control | CUMS | -.00052728325^*^ | 0.000205629 | 0.018 | -0.000953732 | -0.000100835 |
|  |  | taVNS | -2.26774E-05 | 0.000205629 | 0.913 | -0.000449126 | 0.000403771 |
|  | CUMS | Control | .00052728325^*^ | 0.000205629 | 0.018 | 0.000100835 | 0.000953732 |
|  |  | taVNS | .00050460588^*^ | 0.000211591 | 0.026 | 0.000065794 | 0.000943418 |
|  | taVNS | Control | 2.26774E-05 | 0.000205629 | 0.913 | -0.000403771 | 0.000449126 |
|  |  | CUMS | -.00050460588^*^ | 0.000211591 | 0.026 | -0.000943418 | -0.000065794 |
| GABAergic synapse | Control | CUMS | -9.40164E-05 | 5.23485E-05 | 0.086 | -0.000202581 | 1.45477E-05 |
|  |  | taVNS | 6.99024E-05 | 5.23485E-05 | 0.195 | -3.86618E-05 | 0.000178467 |
|  | CUMS | Control | 9.40164E-05 | 5.23485E-05 | 0.086 | -1.45477E-05 | 0.000202581 |
|  |  | taVNS | .00016391875^*^ | 5.38662E-05 | 0.006 | 5.22072E-05 | 0.00027563 |
|  | taVNS | Control | -6.99024E-05 | 5.23485E-05 | 0.195 | -0.000178467 | 3.86618E-05 |
|  |  | CUMS | -.00016391875^*^ | 5.38662E-05 | 0.006 | -0.00027563 | -5.22072E-05 |
| Riboflavin metabolism | Control | CUMS | 0.000217247 | 0.000198769 | 0.286 | -0.000194975 | 0.00062947 |
|  |  | taVNS | .00042799654^*^ | 0.000198769 | 0.043 | 1.57739E-05 | 0.000840219 |
|  | CUMS | Control | -0.000217247 | 0.000198769 | 0.286 | -0.00062947 | 0.000194975 |
|  |  | taVNS | 0.000210749 | 0.000204532 | 0.314 | -0.000213424 | 0.000634923 |
|  | taVNS | Control | -.00042799654^*^ | 0.000198769 | 0.043 | -0.000840219 | -1.57739E-05 |
|  |  | CUMS | -0.000210749 | 0.000204532 | 0.314 | -0.000634923 | 0.000213424 |
| Steroid hormone biosynthesis | Control | CUMS | -.00012792528^*^ | 3.33762E-05 | 0.001 | -0.000197143 | -5.87073E-05 |
|  |  | taVNS | -6.11268E-05 | 3.33762E-05 | 0.081 | -0.000130345 | 8.0912E-06 |
|  | CUMS | Control | .00012792528^*^ | 3.33762E-05 | 0.001 | 5.87073E-05 | 0.000197143 |
|  |  | taVNS | 6.67985E-05 | 3.43438E-05 | 0.065 | -4.4262E-06 | 0.000138023 |
|  | taVNS | Control | 6.11268E-05 | 3.33762E-05 | 0.081 | -8.0912E-06 | 0.000130345 |
|  |  | CUMS | -6.67985E-05 | 3.43438E-05 | 0.065 | -0.000138023 | 4.4262E-06 |
| Tryptophan metabolism | Control | CUMS | -.00073645256^*^ | 0.00027195 | 0.013 | -0.001300443 | -0.000172463 |
|  |  | taVNS | -.00074032481^*^ | 0.00027195 | 0.012 | -0.001304315 | -0.000176335 |
|  | CUMS | Control | .00073645256^*^ | 0.00027195 | 0.013 | 0.000172463 | 0.001300443 |
|  |  | taVNS | -3.87225E-06 | 0.000279834 | 0.989 | -0.000584213 | 0.000576469 |
|  | taVNS | Control | .00074032481^*^ | 0.00027195 | 0.012 | 0.000176335 | 0.001304315 |
|  |  | CUMS | 3.87225E-06 | 0.000279834 | 0.989 | -0.000576469 | 0.000584213 |
