# Supplementary material for: Transcutaneous auricular vagus nerve stimulation improves depressive-like behaviors in CUMS rats through regulation of gut microbiome, serum metabolites, and immune factors
Source: Front Microbiol. 2026 Jul 1;17:1820578. doi: 10.3389/fmicb.2026.1820578 (PMC13369481; doi:10.3389/fmicb.2026.1820578)
Supplement: Supplementary file 6 [file Table_5.DOCX]

***Table S5: One-way ANOVA of colonic short-chain fatty acid concentrations***

| Variables | Total(n=25) | Control (n=9) | CUMS (n=8) | taVNS(n=8) | F-value | p-value |
| --- | --- | --- | --- | --- | --- | --- |
| 2-ECA, Mean±SD | 0.00072±0.00012 | 0.00075±0.00010 | 0.00070±0.00014 | 0.00070±0.00012 | 0.578 | 0.569 |
| HPA, Mean±SD | 0.00408±0.00214 | 0.00426±0.00168 | 0.00348±0.00098 | 0.00448±0.00330 | 0.460 | 0.637 |
| OA, Mean±SD | 0.00539±0.00070 | 0.00502±0.00052 | 0.00541±0.00033 | 0.00578±0.00095 | 2.923 | 0.075 |
| DEA, Mean±SD | 0.00130±0.00056 | 0.00099±0.00030 | 0.00115±0.00058 | 0.00179±0.00047**ab** | 7.120 | 0.004 |
| IBA, Mean±SD | 0.03341±0.01982 | 0.02210±0.00959 | 0.03320±0.02148 | 0.04634±0.02062**a** | 3.941 | 0.034 |
| IVA, Mean±SD | 0.02570±0.01725 | 0.01376±0.00551 | 0.02717±0.01888 | 0.03768±0.01670**a** | 5.740 | 0.010 |
| CA, Mean±SD | 0.03467±0.04539 | 0.04408±0.04230 | 0.02121±0.02509 | 0.03753±0.06371 | 0.540 | 0.591 |
| VA, Mean±SD | 0.05752±0.02797 | 0.04692±0.01311 | 0.05369±0.03605 | 0.07328±0.02723 | 2.189 | 0.136 |
| BA, Mean±SD | 1.36467±0.55657 | 1.24352±0.49577 | 1.50379±0.64896 | 1.36186±0.56483 | 0.442 | 0.649 |
| PA, Mean±SD | 0.83307±0.19336 | 0.84949±0.25405 | 0.83522±0.15974 | 0.81246±0.16756 | 0.072 | 0.930 |
| AA, Mean±SD | 2.53695±0.45378 | 2.77187±0.43997 | 2.42555±0.38995 | 2.38407±0.46995 | 2.071 | 0.150 |
| 4-MVA, Mean±SD | 0.00270±0.00087 | 0.00224±0.00030 | 0.00313±0.00115 | 0.00278±0.00081 | 2.587 | 0.098 |

Note: a indicates significant difference compared to Control group, *P*<0.05; CUMS indicates significant difference compared to CUMS group, *P*<0.05. All pairwise comparisons were adjusted with Bonferroni's correction. 2-ECA, 2-Ethylcaproic acid; HPA, Heptanoic acid; OA, Octanoic acid; DEA, Decanoic acid; IBA, Isobutyric acid; IVA, Isovaleric acid; CA, Caproic acid; VA, Valeric acid; BA, Butyric acid; PA, Propionic acid; AA, Acetic acid; 4-MVA, Isocaproic acid.
